# Supplementary material for: Two-stage optimization based on heterogeneous branch fusion for knowledge distillation
Source: PLoS One. 2025 Jul 2;20(7):e0326711. doi: 10.1371/journal.pone.0326711 (PMC12221028; doi:10.1371/journal.pone.0326711)
Supplement: Attachment — Submitted filename: report.pdf [file pone.0326711.s002.pdf]

Reviewer's report for: *PLOS ONE*
Paper title: Two-stage optimization based on heterogeneous branch fusion for knowledge distillation
Authors: Gang Li, Pengfei Lv , Yang Zhang, Chuanyun Xu, Zihan Ruan, Zheng Zhou, Xinyu Fan, Ru Wang, Pan He

In this paper, the authors investigate knowledge distillation methods and propose a two-stage optimization based on heterogeneous branch fusion for knowledge distillation. The paper is interesting and addresses intriguing questions. The authors conduct experiments rigorously and draw conclusions based on the presented data. While their analysis is thorough and the paper is generally presented in an intelligible manner, it contains ambiguities that hinder readability and discourage further engagement.

- The authors present nine figures in this paper. Unfortunately, the way these figures are presented significantly hinders the readability of the paper. The captions, without the corresponding figures, are scattered throughout the paper, preventing readers from reading the captions while simultaneously viewing the corresponding figures. All nine figures related to these captions are located at the very end of the paper. Moreover, the figure corresponding to the caption 'Fig 1. Two training frameworks. (a) using the OKD method and (b) using the KD method.' is placed as the eighth figure in the list of nine figures located at the end of the paper. Thus, the order of the captions throughout the paper is completely different from the order of the figures at the end. Additionally, the eighth figure in this list contains two parts: '(a) Multi-branch OKD' and '(a) Multi-branch KD', with each part labeled as (a), which is inconsistent with the caption for Figure 1. This should be corrected. Furthermore, each figure should be positioned alongside its caption so that readers can read the figure descriptions while simultaneously viewing the corresponding figures. This will enable readers to easily correlate, for example, the notation used in the captions with the content of the figures. In the current setup, this process is severely hindered. To facilitate reading and encourage engagement with the paper, the authors should reorganize the way their figures are presented.

- The notation used in equations (1), (2), and (3) should be explained in more detail. For example, the authors should clarify the relationship

between z^b and its components z_k^b and z_i^b by defining $z^b = [z_1^b, \ldots, z_K^b]$. Similarly, it would be helpful to define $y = [y_1, \ldots, y_K]$.

- The definitions of $\tilde{p}_k^t$ and $\tilde{p}_k^s$ labeled by (3) are identical. To avoid repetition, the authors should define

$$\tilde{p}_k^j = \frac{\exp(z_k^j/\tau)}{\sum_{i=1}^K \exp(z_i^j/\tau)}, \quad j \in \{t, s\}.$$

- Examples of the fusion function D used in (6) should be provided. Alternatively, references to the literature where such examples are discussed should be included.

- In (6), "$b \in 2, \ldots, B$" should be replaced with $b \in \{2, \ldots, B\}$.
