# Supplementary material for: Two-stage optimization based on heterogeneous branch fusion for knowledge distillation
Source: PLoS One. 2025 Jul 2;20(7):e0326711. doi: 10.1371/journal.pone.0326711 (PMC12221028; doi:10.1371/journal.pone.0326711)
Supplement: Attachment — Submitted filename: Response to Reviewers.pdf [file pone.0326711.s003.pdf]

Dear Editors and Reviewers:

Thank you for your letter and for the reviewers' comments concerning our manuscript. Those comments are valuable and very helpful for revising and improving our paper, as well as the guiding significance to our studies. We have carefully considered the comments and have made revision which is hoped to meet with approval. In order to highlight the changes that we have made, we additionally submit a marked version of the paper and revised portion which is marked in blue.

Reviewer #1:

1.The limitations of existing methods are suggested to be refined to highlight the value of the proposed method. Related work needs to be further organized rather than just listed.

Author response:

Thank you for your review and valuable suggestions. We completely agree with your viewpoint that the original manuscript's analysis of the limitations of existing methods was not sufficiently in-depth, and the organization of related work was somewhat loose. In the revised version, we have conducted a more in-depth analysis of the limitations of existing methods in the related work section and optimized the text accordingly.

2.The contributions of the paper should be stated more clearly and explicitly. The innovation points and contribution statements at the end of the introduction section are suggested to be further refined and condensed to highlight the core values of this paper.

Author response:

Thank you for your review and valuable suggestions. The contribution statement in the original manuscript was somewhat lengthy and did not highlight the core value of this work. In the revised version, we have thoroughly restructured and refined the contribution section at the end of the introduction. The specific modifications are as follows:

(1)We propose a novel knowledge distillation training framework that divides the training process into two stages, enabling the student model to effectively learn comprehensive static knowledge and diverse, dynamic knowledge rather than relying exclusively on the teacher model.

(2)We introduce heterogeneous branches and a Progressive Feature Fusion (PFF) module, which endows the branches with distinct abilities while efficiently fusion differentiated feature representations, thereby enhancing the diversity of dynamic knowledge.

(3)Extensive comparative experiments demonstrate the superior performance of the proposed THFKD across various datasets and network architectures. For instance, on Tiny-ImageNet, THFKD achieves a 0.71% improvement over state-of-the-art (SOTA) methods.

3.More high-quality papers need to be added to further highlight the value of the proposed method, such as the introduction or related work chapters, including but not limited to the following papers:

[1] Knowledge distillation: A survey[J]. International Journal of Computer Vision, 2021, 129(6): 1789-1819.

[2] Learning from Human Educational Wisdom: A Student-Centered Knowledge Distillation Method[J]. IEEE Transactions on Pattern Analysis and Machine Intelligence, 2024, 46(6): 4188-4205.

[3] Improving Knowledge Distillation via Head and Tail Categories[J]. IEEE Transactions on Circuits and Systems for Video Technology, 2024, 34(5): 3465-3480.
and so on…

Author response:

Thank you for your review and valuable suggestions. Following your recommendations, we have added relevant citations in the introduction and related work sections and further optimized the organization of references to more clearly highlight the value of our proposed method.

[1] Knowledge distillation: A survey[J]. International Journal of Computer Vision, 2021, 129(6): 1789-1819.

[2]Xin X, Song H, Gou J. A new similarity-based relational knowledge distillation method[C]//ICASSP 2024-2024 IEEE International Conference on Acoustics, Speech and Signal Processing (ICASSP). IEEE, 2024: 3535-3539.

4.https://github.com/GitLpf/THFKD. The github URL provided by the author is missing the necessary code and other information. However, one of the most valuable contributions of this paper is the availability of their open-source code. Therefore, it is strongly recommended that the authors carefully organize the relevant de-sensitization data, code, and processing flow introduction into github or other open-source forms, and give them in the form of URL links in the Abstract, so as to enhance the influence of the text work and attract more interested readers to follow the work of this paper.

Author response:

Thank you for your recognition and valuable suggestions. We completely agree that the open-source code is one of the key contributions of this work. After receiving your feedback, we have updated the GitHub repository to include the necessary code and data.

5.In the Section of Methodology, the two-stage optimization strategy and progressive feature fusion module should be described in more detail with mathematical formulations.

Author response:

Thank you for your valuable suggestions regarding the methodology section. Based on your feedback, we have provided a detailed mathematical formulation of the two-stage optimization strategy and the progressive feature fusion module in the revised version. The specific additions are as follows:

(1)The feature map of the first branch is used as the initial input fused feature, i.e., $F_1^m = F_1$. The expression for the features outputted by subsequent progressive fusion is:

$$F_b^m = D(F_b, F_{b-1}^m), b \in \{2,..., B\} \tag{7}$$

$$D(F_b, F_{b-1}^m) = w(F_b + F_{b-1}^m) \times F_b + (1-w)(F_b + F_{b-1}^m) \times F_{b-1}^m \tag{8}$$

where $D(\cdot)$ is the fusion function from the Progressive feature fusion module, which is iteratively utilized throughout the training process to progressively amalgamate features. For instance, with the number of branches in the model set to four, the ultimate fused feature is denoted as F_4^m, where the fusion process is articulated as

$$F_4^m = D(F_4, F_3^m) = D(F_4, D(F_3, F_2^m)) = D(F_4, D(F_3, D(F_2, F_1^m))) \quad (2)$$

$$w(i) = e^{-5(1-i/E)^2} \quad (13)$$

where E determines the duration of the weight change period, and i is the i-th epoch with the rate of weight increase being smoothed through the empirical application of a factor of -5. During the training process, the initial parameter a is utilized to regulate the epoch at which the ramp-up weight commences, with the setting range of a being between 0 and 300.

6.In the Section of Methodology, the loss functions and training procedure should be explained more thoroughly.

Author response:

Thank you for your valuable suggestions regarding the methodology section. Based on your feedback, we have modified and added to the loss formula and the training process in the revised version.

7.In the Section of Experiments, The experimental setup (datasets, model architectures, hyper parameters) should be described in more detail.

Author response:

Thank you for your review and valuable suggestions. We have followed the standard experimental setup in the field of knowledge distillation, including the selection of datasets, model architectures, and hyperparameters. To ensure fairness, we have used hyperparameter settings consistent with existing knowledge distillation research, such as temperature coefficient, optimizer, and learning rate scheduling strategies. We have also introduced the relevant literature in the experimental setup section.

8.More ablation studies should be added to analyze the impact of different components (e.g. fusion module, loss weights). Moreover, more tables are suggested to be added to visually present the comparison results of the performance before and after ablation.

Author response:

Thank you for your review and valuable suggestions. In the revised version, we have added ablation experiments on the depth and width of the heterogeneous branches to analyze their impact further. The details are as follows:

To analyze the impact of heterogeneous branches (in terms of width and depth), we conducted experiments on the ResNet32 model by varying the number of auxiliary blocks and the channel size. Specifically, the number of auxiliary blocks was set to 0–2(NA = {0,1,2}), where NA = 0 indicates that each branch has the same structure without auxiliary blocks, and NA = 1 corresponds to the original experimental setting of THFKD. The channel size of the auxiliary blocks was set to either 1× or 2× the number of channels in the high-level layers (CA = { $C\times1$, $C\times2$ }), indicating that each branch has the same capacity. As shown in Table 8, the experimental results demonstrate that the number of auxiliary blocks (NA) and the channel size (CA) impact model performance differently.

First, regarding the number of auxiliary blocks (NA), the homogeneous branches without auxiliary

blocks (NA = 0) exhibited the lowest accuracy (74.3%–74.6%), indicating that the model's feature representation and diversity were constrained. When NA =2, the accuracy of each branch improved (74.8%–75.0%); however, the performance of the more complex Branch-4 was inferior to that of Branch-3. This suggests that continuously increasing the number of auxiliary blocks does not necessarily enhance performance, and an overly complex structure may hinder model optimization. In contrast, the THFKD setting (NA = 1) achieved the best performance (75.0%–76.6%), with branch performance progressively improving, thereby validating the rationality of the heterogeneous branch design.

Regarding the impact of channel size (CA), when the channels were doubled (CA = $C \times 2$), the accuracy of Branch-2 and Branch-3 increased to 75.77% and 75.90%, respectively. However, the accuracy of Branch-1 is similar under both CA = $C \times 1$ and CA = $C \times 2$, indicating that uniform channel expansion (i.e., identical auxiliary blocks) led to branches with similar abilities, failing to capture diverse feature representations. Consequently, the dynamic knowledge lacked sufficient diversity. The performance of THFKD (75.03%–76.61%) further confirms the effectiveness of the heterogeneous branch design.

Table.8 Accuracy(\%) of branches with different abilities on CIFAR-100.

B	Branch-1	Branch-2	Branch-3	Branch-4
NA=0	74.36	74.49	74.33	74.65
NA=2	74.83	75.02	75.64	75.06
CA= $C \times 1$	74.51	74.71	75.16	75.15
CA= $C \times 2$	74.44	75.77	75.90	75.74
THFKD(NA=1)	75.03	75.36	75.20	76.61

Additionally, we have reported ablation experiments on the fusion module and loss weights in the manuscript, which correspond to the loss term L_{el} in Figure 4 and the experimental tables for parameters E and a .

9.The results should be analyzed in more depth, with insights into why the proposed method works well.

Author response:

Thank you for your review and valuable suggestions. We noticed that the analysis of the experimental results in the original manuscript was not sufficiently in-depth. Therefore, in the revised version, we have provided a clearer explanation of the validity of the experimental results. For example, regarding the analysis of Table 5:

We compared the online knowledge distillation methods based on multi-branch models, including CL, ONE, and OKDDip(all configured with four branches during training), with AFID, which employs dual independent models. As shown in Table 5, the first row presents the baseline classification accuracies of the high-parameter models ResNet8x4 and WRN16-2, which are 72.50% and 73.26%, respectively, both higher than the 71.14% accuracy of ResNet32. However, when trained using CL, ONE, and OKDDip, the classification accuracies of these high-parameter models (ResNet8x4 and WRN16-2) decrease compared to ResNet32 (72.56% > 61.81% and 72.43%; 73.50% > 61.66% and 72.86%; 74.32% > 72.75% and 73.96%). Notably, in the AFID,

the independent model training approach does not exhibit such accuracy degradation (74.05% < 74.56% and 74.94%). In contrast, THFKD shows a 1.09% improvement over OKDDip when using ResNet8x4 and a 1.37% improvement over AFID when using WRN16-2 while avoiding the phenomenon where high-parameter models (ResNet8x4 and WRN16-2) underperform ResNet32 in classification accuracy. This demonstrates that THFKD adapts to various model architectures and effectively mitigates the problem of homogenization.

Table4.Compare the results of the OKD method on CIFAR-100.

Student	ResNet32	ResNet8x4	WRN16-2
	71.14	72.50	73.26
CL	72.56	61.81	72.43
ONE	73.50	61.66	72.86
OKDDip	74.32	72.75	73.96
AFID	74.05	74.56	74.94
THFKD	**75.41**	**75.82**	**76.31**

10.In the Section of Conclusion, the conclusion should summarize the key findings and contributions. Limitations of the current work and potential future directions should be discussed.

Author response:

Thank you for your review and valuable suggestions. In the revised version, we have improved the conclusion section to summarize the key findings and contributions more clearly. Additionally, we have added an analysis of the limitations of the current method and discussed potential future research directions to provide a more comprehensive discussion. The specific modifications are as follows:

We propose a novel Two-stage optimization based on heterogeneous branch fusion for knowledge distillation (THFKD). In traditional knowledge distillation, the student model is solely guided by the fixed knowledge of the teacher model, which cannot supplement or expand upon this knowledge, thereby limiting the student's generalization capability. In contrast, our student model leverages heterogeneous branches and a Progressive Feature Fusion (PFF) module to generate collaborative learning objectives, enabling the transfer of diversified, dynamic knowledge rather than relying exclusively on the teacher model. During training, the dynamic knowledge is combined with the static knowledge from the pre-trained teacher, while the two-stage optimization strategy provides appropriate knowledge at different training stages, enhancing the student model's learning efficiency and generalization ability. Extensive experiments were conducted on both standard datasets and long-tailed datasets, demonstrating that THFKD achieves superior performance compared to state-of-the-art methods. This demonstrates its broad application potential in image classification tasks. Additionally, we provide an analysis and visualization to further understand the underlying mechanisms of the proposed method. However, our method also exhibits certain limitations. THFKD performs slightly below existing approaches on lightweight models such as ShuffleNet, and the range of model combinations utilized is not yet comprehensive. In future work, we aim to investigate these limitations further and extend THFKD

to other domains, such as object detection and image segmentation, to explore its broader applicability.

11.The writing should be polished for clarity, grammar, and flow. Some sentences are overly long and could be broken up. Consistent terminology should be used throughout (e.g. "student model" vs "target model").

Author response:

Thank you for your review and valuable suggestions. In the revised version, we have optimized the clarity of the writing to make the expression smoother. Additionally, we have standardized the terminology, such as using "student model" throughout the manuscript instead of "target model," to ensure consistency in the terminology.

Reviewer #2:

1.The authors present nine figures in this paper. Unfortunately, the way these figures are presented significantly hinders the readability of the paper. The captions, without the corresponding figures, are scattered throughout the paper, preventing readers from reading the captions while simultaneously viewing the corresponding figures. All nine figures related to these captions are located at the very end of the paper. Moreover, the figure corresponding to the caption 'Fig 1. Two training frameworks. (a) using the OKD method and (b) using the KD method.' is placed as the eighth figure in the list of nine figures located at the end of the paper. Thus, the order of the captions throughout the paper is completely different from the order of the figures at the end. Additionally, the eighth figure in this list contains two parts: '(a) Multi-branch OKD' and '(a) Multi-branch KD', with each part labeled as (a), which is inconsistent with the caption for Figure 1. This should be corrected. Furthermore, each figure should be positioned alongside its caption so that readers can read the figure descriptions while simultaneously viewing the corresponding figures. This will enable readers to easily correlate, for example, the notation used in the captions with the content of the figures. In the current setup, this process is severely hindered. To facilitate reading and encourage engagement with the paper, the authors should reorganize the way their figures are presented.

Author response:

Thank you for your review and valuable suggestions. I sincerely apologize for the labeling issues you pointed out; this was indeed an oversight on my part. During the preparation of the manuscript, I failed to pay sufficient attention to the details, which caused inconvenience in your reading. I have now revised the figure captions as per your suggestions. Regarding the layout of the figures in the manuscript, I followed the author's guidelines of PLOS ONE when submitting it, but I apologize for the inconvenience this caused. We have submitted a revised version where the figures are presented alongside their captions.

2.The notation used in equations (1), (2), and (3) should be explained in more detail. For example, the authors should clarify the relationship between z^b and its components z_k^b and z_i^b by defining $z^b = [z_1^b, \dots, z_K^b]$. Similarly, it would be helpful to define $y = [y_1, \dots, y_K]$.

Author response:

Thank you for your detailed suggestionson the symbol definitions. We fully agree with your suggestions and have made the necessary revisions. The specific changes are as follows:

Given a dataset with K classes, where $k \in (1,...,K)$, and a training sample x with its corresponding one-hot label $y = [y_1,..., y_K]$, when x is input into the student model, the output probability of the b -th branch for class k is calculated as:

$$p_k^b = \frac{\exp(z_k^b)}{\sum_{i=1}^{K} \exp(z_i^b)} \quad (1)$$

where $z^b = [z_1^b,..., z_K^b]$ is the logit of the \$b\$-th branch. For multi-class classification tasks, training is performed by minimizing the cross-entropy loss between the predicted vector and the ground-truth labels:

3.The definitions of $\tilde{p}_k^t$ and $\tilde{p}_k^s$ labeled by (3) are identical. To avoidrepetition, the authors should define

$$\tilde{p}_k^j = \frac{\exp\left(z_k^j / T\right)}{\sum_{i=1}^{K} \exp\left(z_i^j / T\right)}, j \in \{t, s\}$$

Author response:

Thank you for your detailed suggestions on the definition of the symbol. We fully agree with your suggestions and have made the necessary revisions. The specific changes are as follows:

The core of traditional knowledge distillation (KD) methods is to use the softened teacher outputs as the supervisory signal for training the student model. The formulas for the softened teacher and student output probabilities are as follows:

$$\tilde{p}_k^j = \frac{\exp\left(z_k^j / T\right)}{\sum_{i=1}^{K} \exp\left(z_i^j / T\right)}, j \in \{t, s\} \quad (3)$$

4.Examples of the fusion function D used in (6) should be provided. Alternatively, references to the literature where such examples are discussed should be included.Examples of the fusion function D used in (6) should be provided. Alternatively, references to the literature where such examples are discussed should be included.

Author response:

Thank you for your detailed feedback on the equations. We fully agree with your suggestions and have made the necessary revisions. We have provided specific examples for the fusion function D in equation (6) to more clearly illustrate the process of progressive feature fusion. The specific changes are as follows:

The feature map of the first branch is used as the initial input fused feature, i.e., $F_1^m = F_1$. The expression for the features outputted by subsequent progressive fusion is:

$$F_b^m = D(F_b, F_{b-1}^m), b \in \{2,..., B\} \quad (7)$$

$$D(F_b, F_{b-1}^m) = w(F_b + F_{b-1}^m) \times F_b + (1-w)(F_b + F_{b-1}^m) \times F_{b-1}^m \quad (8)$$

where $D(\cdot)$ is the fusion function from the Progressive feature fusion module, which is iteratively utilized throughout the training process to progressively amalgamate features. For instance, with the number of branches in the model set to four, the ultimate fused feature is denoted as F_4^m, where the fusion process is articulated as $F_4^m = D(F_4, F_3^m) = D(F_4, D(F_3, F_2^m)) = D(F_4, D(F_3, D(F_2, F_1^m)))$.

5.In (6), "b ∈ 2,...,B" should be replaced with b ∈ {2,...,B}.

Author response:

Thank you for your detailed suggestions on the definition of the symbol. We fully agree with your suggestions and have made the necessary revisions. The specific changes are as follows:

$$F_b^m = D(F_b, F_{b-1}^m), b \in \{2,\ldots,B\} \quad (7)$$

Reviewer #3:

1.My main question is in relation to lines 223-235. The text cites Eq. 14, which doesn't exist in this paper, and discusses Eq. 12. It is stated that "In the early stages of training, the student model is susceptible to noise, and the teacher-guided loss term L_{tg} in Eq. 14 dominates. Therefore, the initial weight w(i) is set to a small value and gradually increased over a certain period to allow the teacher to guide the student more smoothly, thereby reducing the impact of noise." However, in the equation both L_{cl} (the collaborative learning loss) and L_{tg} are multiplied by the weight. If multiplying L_{tg} by w(i) was a typographical error, then the article is generally consistent. However, if the equation was written correctly, the authors need to justify their claim that the teacher-guidance dominates at the beginning of training, because this is the whole point of the article. This doubt led to my publication recommendation.

Author response:

Thank you for your review and valuable suggestions. I sincerely apologize for referencing the non-existent equation (14). This was indeed an oversight on our part. During the preparation of the manuscript, we failed to pay sufficient attention to detail, and we have made the necessary corrections in the manuscript.

Regarding equation (12), we confirm that the formatting of the equation in the article is correct. Both the teacher-guided loss L_{tg} and the collaborative learning loss L_{cl} are multiplied by the weight $w(i)$, and this setting is consistent throughout the manuscript. We acknowledge that there was an error in our description, which may have caused confusion in your reading, and we have corrected it in the revised version.

Concerning the statement "teacher guidance dominates in the early stages of training," in the early stages of training, the parameters of the student model have not yet stabilized and are more susceptible to noise. Therefore, we set a smaller initial weight $w(i)$ and gradually increased it to guide the student ' s optimization more smoothly. The main evidence for teacher guidance

dominating in the early stages comes from the loss value curves. From the loss curves in Figure 5, we observe that the teacher-guided loss L_{tg} makes up a larger proportion of the total loss in the early stages, and the difference between the two loss curves in the early stages is significantly larger than in the later stages.Furthermore, since the pre-trained teacher model has stronger performance, a larger number of parameters, and provides more comprehensive and stable knowledge, we believe that teacher guidance dominates in the early stages of training. As training progresses, the student's learning from the teacher becomes saturated, at which point the collaborative learning loss L_{cl} can further optimize the model, enabling it to learn more diverse knowledge.

The corresponding revisions in the manuscript are as follows:

$$L_{total} = \sum_{b=1}^{B} L_{ce}^{b} + w(i)(L_{cl} + L_{tg}) \tag{14}$$

where L_{ce}^{b} is the cross-entropy loss between the b -th branch and the ground-truth label y . During the early stages of training, the parameters of the student model are not yet stabilized and are prone to being affected by noise. Therefore, the initial value of $w(i)$ is set to a relatively small magnitude, and the weight is gradually increased over a predetermined period to guide the student model's optimization smoothly. In Eq. 14, the loss term L_{tg} dominates, enabling the pre-trained teacher model to provide more comprehensive guidance in the initial phases. When $E = i$, the period ends, and the weight is set to 1. At this point, the student has gained a more comprehensive understanding, and diversifying the collaborative learning target becomes more important in the subsequent training. During training, the teacher model's parameters remain unchanged. Taking ResNet32 on CIFAR-100 as an example, the shape of the concatenated logits of all branches is 100×4 (where 4 is the number of branches B), and the loss for each branch is computed along the branch dimension. The performance of the model is primarily evaluated by classification accuracy(%), which represents the proportion of correctly classified samples to the total number of samples across multiple datasets.

Effect of loss in different stages

We analyze the impact of loss at different stages by visualizing the changes in accuracy and loss curves. As shown in Fig5(a), during the early phase (the first 150 epochs), THFKD demonstrates a mean advantage in classification accuracy over the individual use of L_{cl} and L_{tg} for training, with an improvement ranging approximately from 2% to 5%. Furthermore, it can be observed that between epochs 90 and 110, the $L_{tg} + L_{cl}$ curve exhibits more minor fluctuations, with an amplitude of approximately 3%, whereas the fluctuations for L_{cl} and L_{tg} alone are around 12% and 10%, respectively. In Figure5(b), although the loss value of $L_{tg} + L_{cl}$ is approximately

four times that of L_{cl} , the loss value continues to decrease as training progresses, indicating that the teacher's guidance effectively directs the optimization trajectory of the student. Additionally, it can be observed that the L_{tg} loss accounts for a larger proportion of the total loss compared to the L_{cl} loss, suggesting that the teacher's guidance dominates the early gradient updates of the student model. After the learning rate drops at epoch 150, entering the later training stage, the red accuracy curve (L_{cl}) in Fig5(a) is slightly higher than the cyan one (L_{tg}), suggesting that diversified dynamic knowledge further optimizes the student model. However, a reversal occurs in the final stages, which can be attributed to the lack of guidance from the teacher in the early phases. This absence leads to the accumulation of erroneous information within the collaborative learning objectives, and the impact of this phenomenon becomes further amplified as the training progresses into the later stages.

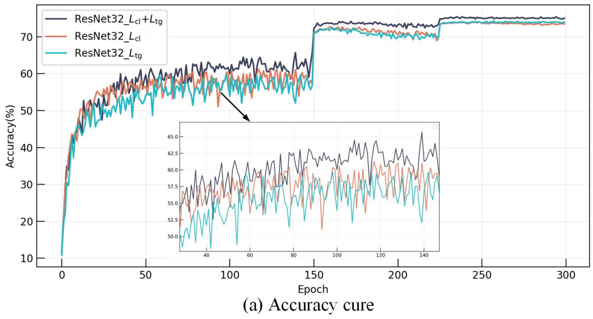

(a) Accuracy cure

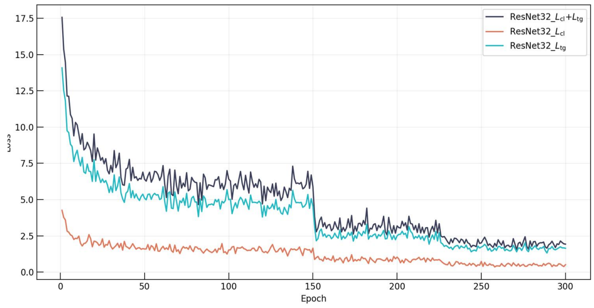

(b) Loss cure

Fig 5. Effect of loss in different stages. (a) Accuracy curves with different losses. (b) Loss value change curves.

2.In the discussion of progressive feature fusion, on line 198, it is not clear if "w" is a scalar or vector. The caption of Figure 3 suggests it's a vector. Please clarify this in the text.Also, the parameter "a" is not introduced during the presentation of the model.

Author response:

Thank you for your review and valuable suggestions. Regarding w in line 198 of the original manuscript, it is a scalar parameter with values ranging from 0 to 1, serving as the global weight for the fused features rather than a vector. We will clarify this point in the revised version and ensure that it is distinguished from the Score in Figure 3 to avoid any ambiguity. Additionally, we have already provided a description of the parameter a in the model introduction section. The specific changes are as follows:

(1)These weight scores are then used to multiply the features F_b and F_{b-1}^m, with parameter g adjusting the importance of each branch, g is a scalar parameter with a value range of 0 to 1, ultimately resulting in the fused features for this level.
(2)During the training process, the initial parameter a is utilized to regulate the epoch at which the ramp-up weight commences, with the setting range of a being between 0 and 300.

3.Lines 305-306: "significantly" should be changed for a word without statistical meaning, perhaps "generally outperforms by an average margin of....". Also, characterize the fluctuations statistically and compare the values.

Author response:

Thank you for your review and valuable suggestions. The term "significant," as a statistical term, is not appropriate in the description on lines 305-306. Therefore, we will revise it to a more suitable expression in the revised version. Additionally, we have provided a more detailed description and numerical comparison of the curve fluctuations in Figure 5(a). The specific changes are as follows:

We analyze the impact of loss at different stages by visualizing the changes in accuracy and loss curves. As shown in Fig5(a), during the early phase (the first 150 epochs), THFKD demonstrates a mean advantage in classification accuracy over the individual use of L_{cl} and L_{tg} for training, with an improvement ranging approximately from 2% to 5%. Furthermore, it can be observed that between epochs 90 and 110, the $L_{tg}+L_{cl}$ curve exhibits more minor fluctuations, with an amplitude of approximately 3%, whereas the fluctuations for L_{cl} and L_{tg} alone are around 12% and 10%, respectively. In Figure5(b), although the loss value of $L_{tg}+L_{cl}$ is approximately four times that of L_{cl}, the loss value continues to decrease as training progresses, indicating that the teacher's guidance effectively directs the optimization trajectory of the student. Additionally, it can be observed that the L_{tg} loss accounts for a larger proportion of the total loss compared to the L_{cl} loss, suggesting that the teacher's guidance dominates the early gradient updates of the student model. After the learning rate drops at epoch 150, entering the later training stage, the red accuracy curve (L_{cl}) in Fig5(a) is slightly higher than the cyan one (L_{tg}), suggesting that diversified dynamic knowledge further optimizes the student model.

4.Lines 308-310: There is an intermediate period where the red line is above the blue line, but this inverts by the end of the period.

Author response:

Thank you for your review and valuable suggestions. In the original manuscript, we did not explicitly describe this phenomenon. However, after further analysis, we found that this reversal

exists, and we have added the corresponding explanation in the revised version. Specifically, we observed that in the final stage of training, the reversal where the red line falls below the blue line occurs due to the lack of teacher guidance in the earlier stages, leading to the accumulation of erroneous information in the collaborative learning objective, which further amplifies in the later stages of training. This phenomenon highlights the critical role of teacher guidance in the early stages to minimize the accumulation of erroneous information. We have provided a more detailed explanation in the revised manuscript to help readers better understand this effect. The specific changes are as follows:

We analyze the impact of loss at different stages by visualizing the changes in accuracy and loss curves. As shown in Fig5(a), during the early phase (the first 150 epochs), THFKD demonstrates a mean advantage in classification accuracy over the individual use of L_{cl} and L_{tg} for training, with an improvement ranging approximately from 2% to 5%. Furthermore, it can be observed that between epochs 90 and 110, the $L_{tg} + L_{cl}$ curve exhibits more minor fluctuations, with an amplitude of approximately 3%, whereas the fluctuations for L_{cl} and L_{tg} alone are around 12% and 10%, respectively. In Figure5(b), although the loss value of $L_{tg} + L_{cl}$ is approximately four times that of L_{cl}, the loss value continues to decrease as training progresses, indicating that the teacher's guidance effectively directs the optimization trajectory of the student. Additionally, it can be observed that the L_{tg} loss accounts for a larger proportion of the total loss compared to the L_{cl} loss, suggesting that the teacher's guidance dominates the early gradient updates of the student model. After the learning rate drops at epoch 150, entering the later training stage, the red accuracy curve (L_{cl}) in Fig5(a) is slightly higher than the cyan one (L_{tg}), suggesting that diversified dynamic knowledge further optimizes the student model. However, a reversal occurs in the final stages, which can be attributed to the lack of guidance from the teacher in the early phases. This absence leads to the accumulation of erroneous information within the collaborative learning objectives, and the impact of this phenomenon becomes further amplified as the training progresses into the later stages.

5.Lines 334-336 (Figure 7): There seems to be an equivalent increase in accuracy from 3 to 4 branches and 4 to 5 branches. Why not use 5 branches?

Author response:

Thank you for your review and valuable suggestions. We have included the relevant explanation in the revised manuscript as follows:

We observed comparable improvements in accuracy when increasing the number of branches from 3 to 4 and from 4 to 5. Although adding branches during testing does not incur additional costs, using 5 branches during training increases computational overhead, including higher GPU memory usage and longer training times, as the number of channels in the auxiliary blocks grows

exponentially. Throughout the experiments, we balanced the performance gains against the computational costs and found that 4 branches were sufficient to achieve substantial performance improvements. Consequently, we selected 4 branches as the optimal configuration for our experimental setup.

---

6. Lines 341-354: Rewrite the text to make it clear which column is being analyzed. Your statements seem general but are only true in one of the columns.

Author response:

Thank you for your review and valuable suggestions. We have rewritten the text in response to the comments on lines 341-354. The previous description was relatively general, so we added more detailed explanations and clarified which data were being compared. The specific changes are as follows:

We compared the online knowledge distillation methods based on multi-branch models, including CL, ONE, and OKDDip(all configured with four branches during training), with AFID, which employs dual independent models. As shown in Table 5, the first row presents the baseline classification accuracies of the high-parameter models ResNet8x4 and WRN16-2, which are 72.50% and 73.26%, respectively, both higher than the 71.14% accuracy of ResNet32. However, when trained using CL, ONE, and OKDDip, the classification accuracies of these high-parameter models (ResNet8x4 and WRN16-2) decrease compared to ResNet32 (72.56% > 61.81% and 72.43%; 73.50% > 61.66% and 72.86%; 74.32% > 72.75% and 73.96%). Notably, in the AFID, the independent model training approach does not exhibit such accuracy degradation (74.05% < 74.56% and 74.94%). In contrast, THFKD shows a 1.09% improvement over OKDDip when using ResNet8x4 and a 1.37% improvement over AFID when using WRN16-2 while avoiding the phenomenon where high-parameter models (ResNet8x4 and WRN16-2) underperform ResNet32 in classification accuracy. This demonstrates that THFKD adapts to various model architectures and effectively mitigates the problem of homogenization.

Table4.Compare the results of the OKD method on CIFAR-100.

Student	ResNet32	ResNet8x4	WRN16-2
	71.14	72.50	73.26
CL	72.56	61.81	72.43
ONE	73.50	61.66	72.86
OKDDip	74.32	72.75	73.96
AFID	74.05	74.56	74.94
THFKD	**75.41**	**75.82**	**76.31**

7.Lines 375-379: Is it surprising that the branches with more complex architectures (because of the increasingly complex auxiliary blocks) have higher accuracies?

Author response:

Thank you for your review and valuable suggestions. Our analysis is not unexpected Regarding whether branches with more complex architectures achieve higher accuracy. The results

mentioned in the text indicate that its performance improves as the branch architecture becomes increasingly complex (due to the gradual increase in the number of channels in the auxiliary blocks). The reason for this improvement is that a more complex architecture can better process different features, enhancing the overall representational capacity of the model. We believe this is reasonable because, as the architectural complexity increases, the model can learn more fine-grained details, improving accuracy. We also emphasize that this finding lays the foundation for diverse collaborative learning objectives while enabling THFKD to meet varying accuracy requirements and provide more flexible deployment options.

8.Figure 1 labels both panels as 1a.

Author response:

Thank you for your correction. We have updated the labels in Figure 1, marking the two panels as 1a and 1b to ensure clear differentiation.

9.Line 161: I suggest writing something like, "to the target branch structure" instead of "to the target branch"?

Author response:

Based on your suggestion, we have revised "to the target branch" to "to the target branch structure" to make the expression more accurate and clear.

10.Line 226: "Equation (14)" should be "Equation (12)".

Author response:

Thank you for your thorough review of our paper. We have corrected the equation references to ensure consistency with the text.

11.Line 248: The description of the number of images in Tiny-ImageNet is a little confusing. Try to explain this part more clearly.

Author response:

Thank you for your thorough review and valuable suggestions. We have clarified the description of the number of images in Tiny-ImageNet to ensure accuracy and improve clarity. The specific changes are as follows:

The Tiny-ImageNet dataset contains 200 classes with 20,000 RGB images of size 64×64. It includes 100,000 training images and both 10,000 testing and 10,000 validation images.

12.Line 292: Wrong equation reference.

Author response:

Thank you for your thorough review and valuable suggestions. We have reviewed and corrected the incorrect equation references:

Different combinations of loss impact classification accuracy, as shown in Eq. 11: $L_{cl} = L_{fl} + L_{el}$.

13.Figure 5: "Loss cure" should be "Loss curve".

Author response:

Thank you for your thorough review and valuable suggestions. We have corrected "Loss cure" to the correct term "Loss curve."

14.Line 313: Correct the English.

Author response:

Thank you for your thorough review and valuable suggestions. We have revised the English expression in line 313 to make it clearer and more accurate:

By controlling the period parameter E and a the starting parameter, we evaluated the impact of the weight increase period and the two-stage optimization strategy on the model performance.

15.Line 372: Clarify this sentence.

Author response:

Thank you for your thorough review and valuable suggestions. We have revised the expression in line 372 to ensure the sentence is more apparent. The specific changes are as follows:

Ensembling the predictions of multiple branches can lead to stronger performance. As shown in Table\ref{table6}, we retain three branches for the ensemble output (THFKD-E). Benefiting from the increased diversity, THFKD-E achieves a more effective ensemble. \replaced{When the number of parameters in the ensemble model is less than or equal to that of the teacher model, the ensemble's predictions achieve higher accuracy than those made by the teacher model.}{When the parameter is less than or equivalent to the teacher model, the ensemble predictions achieve higher accuracy than the teacher model.

Table6.Ensemble prediction Accuracy(\%) and parameters(MB) of THFKD with three branches on CIFAR-100.

Teacher	ResNet56(0.86 MB)	ResNet110(1.71 MB)	ResNet32x4(7.43 MB)	WRN40-2(2.25 MB)	WRN40-2(2.25 MB)
	72.34	74.31	79.42	75.61	75.61
Student	ResNet20	ResNet32	ResNet8x4	WRN16-2	WRN40-1
	69.06	71.14	72.50	73.26	71.98
THFKD	72.76 (0.39MB)	74.51 (0.58MB)	75.12 (1.23MB)	76.51 (0.70MB)	75.24 (0.56MB)
THFKD-E	76.29 (0.85MB)	78.05 (1.40MB)	80.21 (6.46MB)	79.31 (2.62MB)	78.33 (1.64MB)

16.Figure 9: "Branch", not "Branach".

Author response:

Thank you for your reminder. We have corrected "Branch" to the correct term, "Branch."

17.Line 393: Clarify this sentence.

Author response:

Thank you for your thorough review and valuable suggestions. We have revised the expression in line 393 to ensure the sentence is more apparent. The specific changes are as follows:

This study uses Grad-CAM to visualize the feature regions attended to by the model, as shown in Figure9, including the heatmaps of each branch and PFF-3, along with the ground truth labels and predicted classes. Columns 1 and 2 show the cases where each branch and PFF-3 classify correctly, demonstrating high certainty for simple images. In Column 2, the larger-capacity branches focus more on local features. In Column 3, although each branch and PFF-3 focus on the correct regions, they predict three different categories. This demonstrates that the student can learn more comprehensively by softening logits to transfer similar information between categories and combining both static and dynamic knowledge. Only Branch-4 can correctly predict the class in the fourth column, demonstrating that THFKD can further optimize the student model in the later stages. We also observe that the feature heatmap of PFF-3, compared to those of Branches 1-4, captures more accurate feature information, indicating that PFF effectively fuses the differentiated feature representations.

18.Line 412: "further to" -> "to further".

Author response:

Thank you for your thorough review and valuable suggestions. We have corrected "further to" to the proper phrasing "to further" to ensure grammatical accuracy.

Reviewer #4:

1.*The heterogeneous branch structure and progressive feature fusion module are central to the proposed approach, yet the theoretical foundation behind their effectiveness is not fully explored. A deeper mathematical discussion on how heterogeneous branches enhance student model learning would strengthen the study.*

Author response:

(1)Thank you for your review and valuable suggestions. We understand your concerns regarding the theoretical basis of the heterogeneous branch structure and the Progressive Feature Fusion (PFF) module. In the revised version, we have further discussed the theoretical foundation of the heterogeneous branches and provided a more in-depth analysis of how they enhance the student model. The specific modifications are as follows:

Let F_b^C denote the feature map input to the auxiliary block, where C represents the number of channels in the feature map. The specific formulation is as follows:

$$F_b^{C_A} = Conv1(F_b^C),\ F_b^{C_A} = Conv2(F_b^{C_A}),\ F_b = Conv2(F_b^{C_A}) + F_b^C \quad (6)$$

Where $Conv1$ is employed to increase the number of channels($C - C_A$), $Conv2$ extracts higher-level feature information through the expanded channels, and $Conv3$ uniformly restores the channel count to C . The output is then combined with the original feature map F_b^C via a residual connection to mitigate overfitting.Auxiliary blocks with a gradually increasing number of channels are added to the branches, creating differences in receptive field expansion and feature dimensions. This design forces different branches to learn complementary feature representations: shallow branches learn the fundamental features of samples, while deep branches capture subtle differences.

(2)In the revised version, we have further discussed the theoretical foundation of the Progressive Feature Fusion (PFF) module. The specific modifications are as follows:

We propose heterogeneous branches that differ from the target branch regarding model depth and feature representation. By introducing a progressive feature fusion module, we gradually combine the differentiated feature representations progressively, thereby enhancing the diversity of dynamic knowledge.

The feature map of the first branch is used as the initial input fused feature, i.e., $F_1^m = F_1$ The expression for the features outputted by subsequent progressive fusion is:

$$F_b^m = D(F_b, F_{b-1}^m), b \in (2,...,B) \tag{7}$$

$$D(F_b, F_{b-1}^m) = w(F_b + F_{b-1}^m) \times F_b + (1-w)(F_b + F_{b-1}^m) \times F_{b-1}^m \tag{8}$$

where $D(\cdot)$ is the fusion function from the Progressive feature fusion module. which is utilized iteratively throughout the training process to amalgamate features in a progressive manner. For instance, with the number of branches in the model set to four, the ultimate fused feature is denoted as F_4^m, where the fusion process is articulated as $F_4^m = D(F_4, F_3^m) = D(F_4, D(F_3, F_2^m)) = D(F_4, D(F_3, D(F_2, F_1^m)))$.

Considering that the focus of this study is on experimentally validating the effectiveness of the method, the mathematical discussions are indeed somewhat limited. We will strengthen this aspect in future work. Thank you again for your valuable suggestions.

2.While the experiments are comprehensive, further analysis of branch heterogeneity (e.g., varying branch widths, depths) is needed to isolate its impact.

The study should also evaluate the model's performance with and without the PFF module to clarify its contribution.

Extending experiments to additional tasks (e.g., object detection, segmentation) would help assess generalization beyond classification.

Author response:

(1)Thank you for your valuable suggestions regarding the experimental design. Based on your feedback, we have included the following analysis of branch heterogeneity in the revised version:

To analyze the impact of heterogeneous branches (in terms of width and depth), we conducted experiments on the ResNet32 model by varying the number of auxiliary blocks and the channel size. Specifically, the number of auxiliary blocks was set to 0 – 2 (NA={0,1,2}), where NA = 0 indicates that each branch has the same structure without auxiliary blocks, and NA = 1 corresponds to the original experimental setting of THFKD. The channel size of the auxiliary blocks was set to either 1 × or 2 × the number of channels in the high-level layers (CA= ($C \times 1$, $C \times 2$)), indicating that each branch has the same capacity. As shown in Table 8, the experimental results demonstrate that the number of auxiliary blocks (NA) and the channel size (CA) have different impacts on model performance.

First, regarding the number of auxiliary blocks (NA), the homogeneous branches without auxiliary blocks (NA=0) exhibited the lowest accuracy (74.3% – 74.6%), indicating that the model's feature representation and diversity were constrained. When NA=2, the accuracy of each branch improved (74.8% – 75.0%); however, the performance of the more complex Branch-4 was inferior to that of Branch-3. This suggests that continuously increasing the number of auxiliary blocks does not necessarily enhance performance, and an overly complex structure may hinder model

optimization. In contrast, the THFKD setting (NA=1) achieved the best performance (75.0% – 76.6%), with branch performance progressively improving, thereby validating the rationality of the heterogeneous branch design.

Regarding the impact of channel size (CA), when the channels were doubled (CA= $C\times2$), the accuracy of Branch-2 and Branch-3 increased to 75.77% and 75.90%, respectively. However, the accuracy of Branch-1 similar under both CA= $C\times2$ and CA= $C\times1$, indicating that uniform channel expansion (i.e., identical auxiliary blocks) led to branches with similar abilities, failing to capture diverse feature representations. Consequently, the dynamic knowledge lacked sufficient diversity. The performance of THFKD (75.03\% – 76.61\%) further confirms the effectiveness of the heterogeneous branch design.

Table8. Accuracy of Branch 1-4 with heterogeneous branches at different depths and widths on CIFAR-100.

B	Branch-1	Branch-2	Branch-3	Branch-4
NA=0	74.36	74.49	74.33	74.65
NA=2	74.83	75.02	75.64	75.06
CA= $C\times1$	74.51	74.71	75.16	75.15
CA= $C\times2$	74.44	75.77	75.90	75.74
THFKD(NA=1)	75.03	75.36	75.20	76.61

(2)Thank you for your valuable feedback. In our method, the Progressive Feature Fusion (PFF) module plays a crucial role in the learning process of the student model, which is why we compared the cases with and without the PFF module in our experiments. The experiment in Figure4 specifically validates this point. Without the PFF module, the model cannot perform feature fusion, and as a result, the loss L_{el} cannot be computed. We have clearly demonstrated the performance differences with and without the PFF module in the experiment and highlighted the positive contribution of the PFF module to the model' s performance. We believe that this setup helps clarify the performance implications of having or not having the PFF module.

(3)We completely agree that extending the experiments to tasks like object detection and segmentation would further enhance the generality of our method. Due to time and resource constraints, we plan to extend this work in the future, for example, by validating performance on object detection tasks using the COCO datasets and on semantic segmentation tasks using the Cityscapes datasets.

3.A sensitivity analysis on the ramp-up weight period (E) and temperature parameter (τ) would be valuable.

Results with different teacher architectures (e.g., ViTs, EfficientNets) could offer additional insights.

Author response:

Thank you for your review and valuable suggestions. Table 4 presents the ablation study on the weight ramp-up period (E). Additionally, we have added an ablation study on the temperature

parameter (τ), with the specific modifications as follows:

Impact of temperature parameter τ

The temperature parameter τ has a notable impact on the performance of the THFKD method. As shown in Table 5, the highest accuracy (75.41%) is achieved when $\tau = 3$, making it the optimal setting. A lower temperature ($\tau = 2$) may result in insufficient soft target information, limiting the learning capability of the student model. On the other hand, as the temperature increases to $\tau = 4$ and $\tau = 5$, the performance gradually declines, indicating that excessively high temperatures may overly smooth the probability distribution, leading to the loss of critical information. Therefore, $\tau = 3$ represents a balanced temperature that maintains sufficient information while avoiding excessive smoothing, providing effective guidance for the student model.

Table 5. Effect of temperature parameter τ

τ	2	**3**	4	5
THFKD	74.29	**75.41**	74.93	74.72

(2) Regarding using "different teacher architectures," to ensure fairness and comparability, we followed the model architectures commonly used in mainstream knowledge distillation research. This ensures that comparisons with existing methods are conducted under the same conditions. We acknowledge that exploring different teacher architectures is a valuable direction for future work. However, due to the high training costs of models such as ViTs, we plan to investigate this aspect further in our future research.

Once again, we sincerely appreciate your valuable feedback.

4.The figures should be better annotated, particularly Figures 2 (multi-branch structure) and 3 (PFF module).

Some terminology is used interchangeably (e.g., "auxiliary blocks" vs. "auxiliary branches"), which could be standardized for better readability.

Author response:

Thank you for your valuable suggestions regarding the clarity of the figures and consistency of terminology. Based on your feedback, we have made the following improvements in the revised version:

(1)We have modified the labels and symbols in Figure 2 (Multi-branch Structure) and Figure 3 (PFF Module).

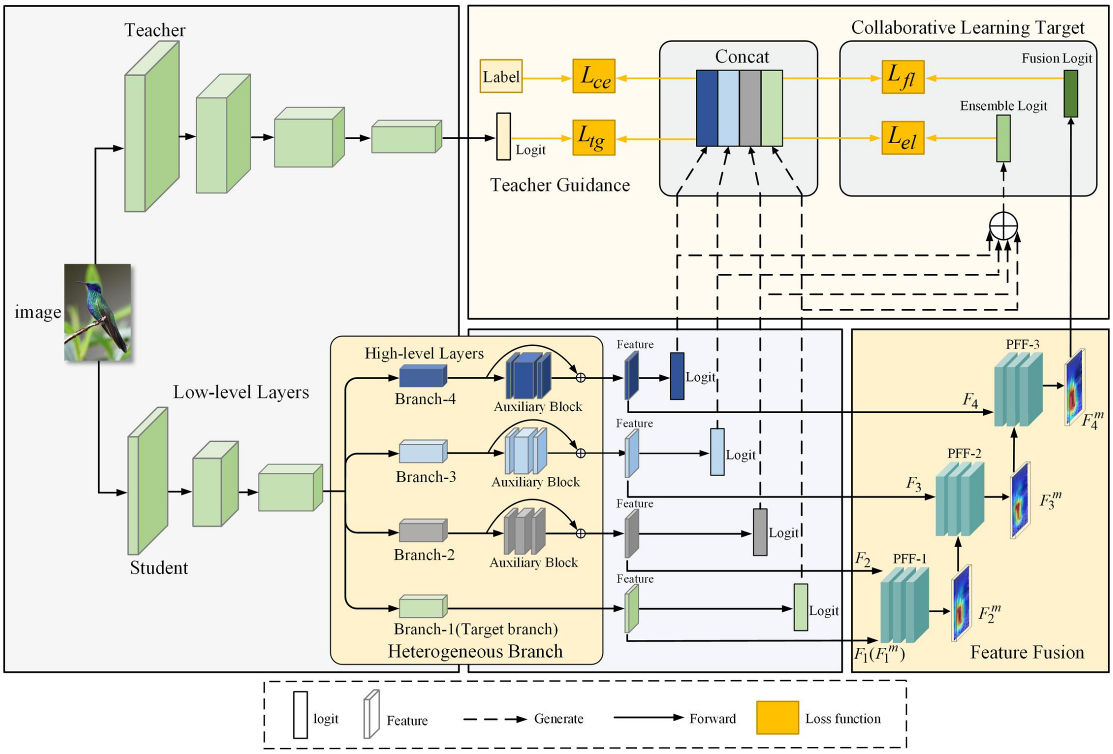

Figure 2

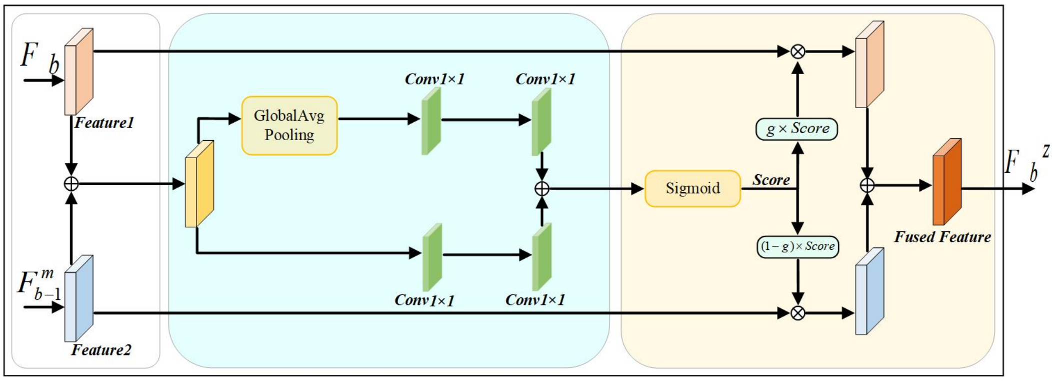

Figure 3

The term "auxiliary block" is now consistently used to refer to the additional structures in the heterogeneous branches, avoiding confusion with "auxiliary branch."

We have ensured consistent use of terminology for "student model" and "target model" throughout the manuscript.

5.The study discusses knowledge distillation and online distillation but does not compare its approach with recent hybrid distillation methods (e.g., semi-online KD, ensemble-based KD). The authors should clarify how their method differs from or improves upon Snapshot Distillation and Focal Distillation.

Author response:

Thank you for your valuable suggestions regarding the comparison with related work. We fully understand your concerns about the method comparison, and we would like to address the issues you raised as follows:

(1)We have observed that current semi-online knowledge distillation and ensemble KD methods differ significantly in network architecture from the mainstream structure used in our work. For

example, semi-online KD methods often adopt specific network structures when constructing teacher-student models, while ensemble-based KD generally requires additional model parameters and computational resources, affecting fairness and practical applicability. To ensure a fair and practical comparison, we referenced the network architecture of SOTA methods in our research, ensuring that we validated the effectiveness of the online distillation method without introducing extra parameters. Our method improves model performance without relying on specialized network architectures, which provides a clear advantage in practical applications.

(2)Snapshot distillation uses snapshots of the teacher model at different training stages for knowledge transfer. This method divides the training process into several stages, each involving a learning rate decay. In contrast, our method utilizes the firm guidance of a pre-trained teacher, adjusting only the weight of the loss function without changing parameters such as the learning rate. While snapshot distillation is a pioneering approach, subsequent research on it has been relatively limited, and its performance has not been competitive with current SOTA methods. Focused distillation primarily targets object detection tasks by focusing on complex samples to improve detection performance. Our research focuses on image classification tasks, so object detection is not directly involved in the current experimental setup. We recognize the advantages of focused distillation in object detection. We plan to extend our method to the object detection domain in future work, where we will perform a comprehensive comparison with focused distillation.

Reviewer #5:

I indicated that the statistical analysis has not been performed appropriately b cause the authors refer several times to a "significant difference" or "significant improvement" but no proper statistical analysis justifying such significance is performed. Following are the places of the manuscript where the word "significant" is used:

1) Abstract, Lines 70-71, Lines 405-406: "THFKD significantly improves the student model's classification accuracy".

2) Lines 56-57: "Experimental results show that the integration of multiple branches can significantly improve performance."

3) Lines 266-267: "Different architectures significantly impact knowledge distillation performance."

4) Line 291: "Different combinations of loss significantly impact classification accuracy".

5) Lines 305-306: "THFKD(Lcl + Ltg) significantly outperforms the accuracy of models trained with Lcl or 305 Ltg individually".

6) Lines 334-335: "As shown in Fig7, THFKD exhibits significant performance improvement when the number of branches is set to 4."

7) Line 338: "When the number of branches is significantly increased". --> remove the word "significantly" here as the number of branches takes the values 1, 2, 3, 4, 5.

8) Line 348: "using the CL, ONE, and OKDDip methods significantly decreased".

My recommendation is to either:

a) Provide a statistical analysis proving that the differences and improvements mentioned are indeed significant, e.g. by computing confidence intervals for the plotted curves and bars.

b) Defining precisely what is meant by "significant". Perhaps it is given by a change in the number of correctly classified images above a pre-defined threshold?

c) Remove the word "significant" from all the aforementioned sentences.

Author response:

Thank you for your detailed review and valuable suggestions. The issue you raised regarding statistical analysis is very important. We have carefully considered your feedback and have removed the word "significant" from all the sentences mentioned above.

MINOR comments:

1.Please make all vertical axes start at 0 in ALL plots, in order to avoid a misinterpretation of the magnitude of the differences among the methods compared. This is specially apparent in Figures 4 and 7.

Author response:

Thank you for your detailed review and valuable suggestions. Regarding the issue you raised about the y-axis not starting from 0 in the figures, we understand your concerns and would like to explain. In Figures 4 and 7, we did not primarily start the y-axis from 0 to more clearly display the accuracy differences under different settings. Since the accuracy values of the experimental results are concentrated within a narrow range, starting the y-axis from 0 would make the differences appear very small and difficult to observe intuitively. This design choice is common in research in related fields and aims to highlight the key differences in experimental results more effectively. Thank you again for your valuable feedback.

2.Please define how model performance is measured.

Author response:

Thank you for your detailed review and valuable suggestions. Regarding the issue you raised about how to measure model performance, we take this matter seriously and have further clarified the relevant content in the revised manuscript:

The model's performance is primarily evaluated by classification accuracy(%), which represents the proportion of correctly classified samples to the total number of samples across multiple datasets.

3.Please clarify what is shown in the tables. Instead of using "Results of..." use e.g. "Accuracy on test sets of...".

Author response:

Thank you for the valuable suggestions provided by the reviewer. We completely agree that the metric descriptions in the tables should be clearer and more specific. Following your recommendation, we have revised the headers of all relevant tables and replaced the original term 'Results' with 'Accuracy (%)' to ensure clarity and accuracy in the presentation of the data.

4.Please define "capacity". E.g. in "high-capacity models" which is used a couple of times.

Author response:

Thank you for your valuable suggestion. To avoid any ambiguity with the term 'capacity,' we have revised the entire manuscript and replaced the 'high-capacity model' with the 'high-parameter model.' In this study, the term 'model parameter count' refers to the total number of trainable parameters in the model, including convolutional kernel weights, biases, and so on.

MAJOR COMMENTS:

1 The definition of the cross-entropy loss should be corrected. Currently:

a) the summation should be over the samples, in addition to the classes.

b) the label "y" is not fully defined for a correct interpretation of the cross-entropy loss. In fact, labels can be anything, they don't even need to be numbers. Please consider mentioning the one-hot encoding of the labels or the use of the indicator function applied on "y(i)" of the true label "k" of each sample "i".

c) a minus sign is missing, which states that the objective is to minimize the loss, not maximize it (in fact, the loss should achieve its minimum of zero when the estimated probability for a sample in class k of being in class k is equal to 1.0).

Author response:

Thank you for your thorough review and valuable suggestions. Your comments regarding the definition of the cross-entropy loss are very important, and we will make the following revisions to the relevant content in the revised manuscript:

$$L_{ce}^{b} = -\sum_{i=1}^{N}\sum_{k=1}^{K} y_{i,k} \log p_{i,k}^{b} \tag{2}$$

where N represents the number of samples, $y_{i,k}$ is the ground-truth label (one-hot encoded) of sample i, and $p_{i,k}$ is the predicted probability by the model that sample i belongs to class k.}

---

2 Fig. 3:

a) The fused features from the previous level is called F^m_{b-1} in the text and F^z_{b-1} in the figure. Please unify notation. Also, what is `m`?

b) Please indicate that BN = Batch Normalization and ReLU is "Rectified Linear Unit" (e.g. as a footnote).

Author response:

(1)Thank you for your thorough review and valuable suggestions. To ensure consistency, we have unified the notation to F_{b-1}^{m}. Superscript m denotes the fused feature after the multi-branch fusion process.

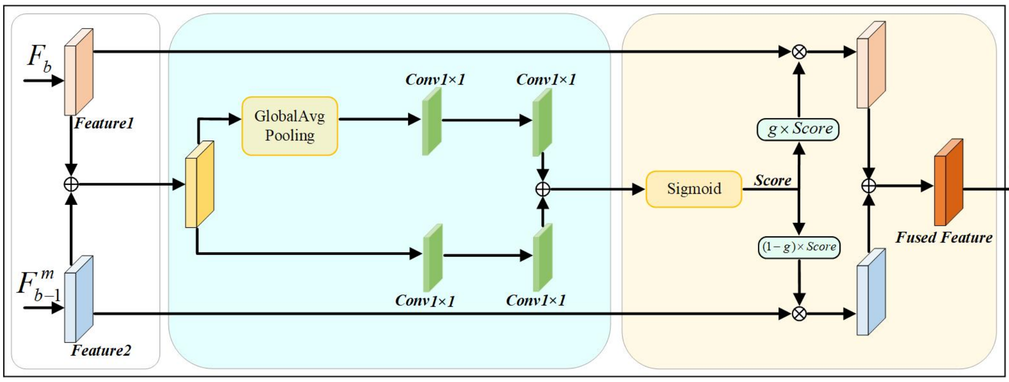

(2)We have removed the explicit annotations of BN and ReLU from Figure 3, as they are standard components and do not require additional emphasis. This adjustment ensures that the figure remains clear and focused on the key aspects of the architecture.

---

3 Line 269 reads "...THFKD improves by 0.27% with ResNet50-MobileNetV2...", but:

a) the value 0.27% is not correct as the difference on the rightmost column of 70.02 - 69.89 = 0.23;

b) the difference between THFKD and ReviewKD for ResNet32x4-ShuffleNetV2 is 77.51 - 77.78 = -0.27.

Hence, I don't find it fair to write "THFKD improves by ... with ResNet50-MobileNetV2" while writing "achieves comparable performance" when talking about the ResNet32x4-ShuffleNetV2 architecture, as their differences are about the same but with different sign (0.23 vs. -0.27, respectively).

Therefore, I encourage the authors to write "THFKD and ReviewKD achieve comparable performance with ResNet50-MobileNetV2 and ResNet32x4-ShuffleNetV2".

Author response:

Thank you for your careful review and valuable feedback. We sincerely appreciate your attention to detail and agree with your observations regarding the performance comparisons between THFKD and ReviewKD. We apologize for the oversight in calculating THFKD's performance improvement on ResNet50-MobileNetV2.

We agree with the reviewer that the performance differences between THFKD and ReviewKD on ResNet50-MobileNetV2 (0.23%) and ResNet32x4-ShuffleNetV2 (-0.27%) are nearly identical in magnitude, differing only in sign. We have revised the text to reflect this observation and ensure fairness and accuracy in our description. Specifically, "THFKD and ReviewKD achieved comparable performance on ResNet50-MobileNetV2 and ResNet32x4-ShuffleNetV2."

This revision eliminates any potential bias in interpreting the results and provides a more balanced and objective comparison.

Thank you again for your constructive comments, which have helped us improve the clarity and accuracy of our manuscript.

---

4 Lines 254-259: Please provide a justification for the selection of the different hyperparameters, or a reference that suggests those choices.

Author response:

Thank you for your detailed review and valuable suggestions. Regarding the issue you raised about the hyperparameter selection in lines 254-259, to ensure fairness and comparability, we followed the hyperparameter settings and model architectures commonly used in mainstream knowledge distillation research. This approach ensures that our comparisons with existing methods are conducted under the same conditions. Therefore, we selected reasonable settings based on existing literature and have cited the relevant references in the revised manuscript.

---

5 Line 312: "Effect of two-stage optimization"

Consider improving this subsection. The following points make the description unclear:

a) The starting parameter "a" is mentioned in the first paragraph but only defined in the second

paragraph.

b) The starting parameter "a" is sometimes written in italics (correct) and sometime writen in regular typescript (incorrect, as it gets confused with the undetermined English article "a").

c) The first paragraph starts "By controlling the period parameter E and a the starting parameter a, ...", i.e. "a" is incorrectly used twice, generating confusion.

d) In the second paragraph we read the following phrase: "the accuracy sharply increases in the current epoch". What is "current epoch"? It would be clearer if you wrote: "the accuracy sharply increases

at the epoch 'a' when the ramp-up weight starts being used".

e) Fig. 6's caption should read rather: "Impact of parameter 'a'"..

Author response:

Thank you for your detailed review and valuable suggestions. We fully agree with your viewpoint and have made the following revisions to the relevant content in the revised manuscript:

By controlling the period parameter E and a the starting parameter, we evaluated the impact of the weight increase period and the two-stage optimization strategy on the model performance. As shown in Table 4, when the period length is controlled during the early stage of training (before the learning rate decreases at epoch 150), the impact on model accuracy is minimal. However, when the period extends into the later stages of training, the change in weight loss is detrimental to the convergence of the student model, leading to a significant drop in accuracy. When E is 0 or 300 (i.e., a single-stage optimization strategy), the loss weight gradually increases or remains constant throughout the training process, resulting in the classification accuracy of THFKD being lower than that obtained with the two-stage optimization strategy.

Table 4. The Impact of the period parameter E

E	0	40	80	120**(THFKD)**	160	300
Accuracy	74.71	75.14	75.23	**75.41**	74.64	74.70

In the training phase, the starting parameter a controls at which epoch THFKD begins to use ramp-up weights. As shown in Fig 6, when $a = 0$, the accuracy curve of the model steadily rises, indicating that THFKD completes the early learning phase within 80 epochs, laying the foundation for subsequent training, thereby maintaining the highest accuracy. However, when a is set to 30, 60, or 90, the accuracy sharply increases at epoch= a (when the ramp-up weight is first applied) but then drops immediately, suggesting that changing the loss weight mid-training disrupts the optimization of the model. Therefore, by using ramp-up weight at the beginning of training and setting the period length to 120, THFKD achieves the best two-stage optimization strategy.

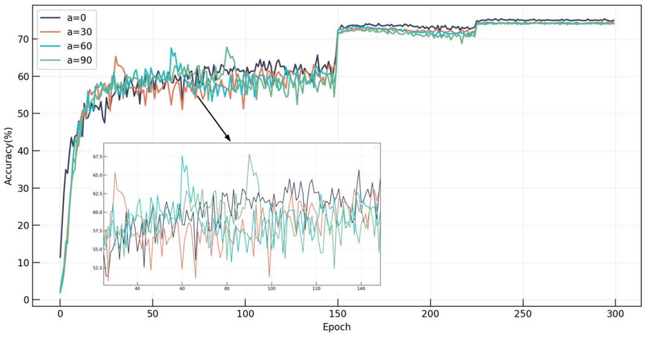

Fig 6. Impact of the parameter a .

6 Lines 406-410: The sentences describing the experimental results should be improved. Specifically:

a) What is the "with ResNet32 and ResNet100" case? Perhaps you meant the "ResNet100-ResNet32" case corresponding to column 2 in Table 1?

b) It would help to have the table numbers from which the results are taken for being able to easily fetch the results and understand better (e.g. "see column 2 in Table 1").

c) Reporting the absolute accuracy doesn't help much in understanding the benefits of the proposed method. The change in accuracy should also be reported w.r.t. to what is considered to be the SOTA method.

d) Why are those results selected for reporting in the Conclusions? Is it because the ResNet32 is considered to be a commonly used architecture? If this is the case, please indicate so.

e) Why is the THFKD with accuracy 58.1% chosen for reporting instead of the case with accuracy 60.7% shown in Table 3?

f) The final sentence should read "...we provide an analysis and visualization to further understand..." instead of "...further to...".

Author response:

Thank you for your detailed review and valuable suggestions. We have carefully revised the relevant content to enhance clarity and rigor. We apologize for the oversight regarding ResNet32 and ResNet100 in the text, which should have been ResNet32 and ResNet110. Taking your suggestions into account, we believe that directly providing the result sources and reporting absolute accuracy does not significantly help in understanding the advantages of the proposed method. We have rewritten the conclusion section by referencing the structure of high-quality literature:

We propose a novel Two-stage optimization based on heterogeneous branch fusion for knowledge distillation (THFKD). In traditional knowledge distillation, the student model is solely guided by the fixed knowledge of the teacher model, which lacks the ability to supplement or expand upon knowledge, thereby limiting the student's generalization capability. In contrast, our student model leverages heterogeneous branches and a Progressive Feature Fusion (PFF) module to generate collaborative learning objectives, enabling the transfer of diversified dynamic knowledge, rather than relying exclusively on the teacher model. During training, the dynamic knowledge is combined with the static knowledge from the pre-trained teacher, while the two-stage optimization strategy provides appropriate knowledge at different training stages, enhancing the student model's learning efficiency and generalization ability. Extensive experiments were conducted on both standard datasets and long-tailed datasets, demonstrating that THFKD achieves superior performance compared to state-of-the-art methods. This demonstrates its broad application potential in image classification tasks. Additionally, we provide an analysis and visualization to further understand the underlying mechanisms of the proposed method. However, our method also exhibits certain limitations. THFKD performs slightly below existing approaches on lightweight models such as ShuffleNet, and the range of model combinations utilized is not yet comprehensive. In future work, we aim to further investigate these limitations and extend THFKD to other domains, such as object detection and image segmentation, to explore its broader applicability.

MINOR COMMENTS:

1 To simplify reading and avoid having to refer to the references section every time the reader encounters an acronym, please mention the full name of the acronym the first time the acronym is used. This happens several times (~10) in the manuscript (e.g. DML, ONE, CKA, FFL, AFF, etc.)

Author response:

Thank you for your detailed review and valuable suggestions. Regarding the issue of abbreviations, we fully agree with your opinion and have made the following revisions to the revised manuscript. Specifically, we have provided the full terms upon the first use of each abbreviation, as follows:

Deep Mutual Learning (DML)
On-the-Fly Native Ensemble (ONE)
Similarity of Neural Network Representations Revisited (CKA)
NRKD (A New Similarity-Based Relational Knowledge Distillation)
ReviewKD (Via Knowledge Review)
OKDDip (Online Knowledge Distillation with Diverse Peers)
PCL (Peer Collaborative Learning)
FFL (Feature Fusion for Online Mutual Knowledge Distillation)
AFF (Attentional Feature Fusion)

These modifications will help readers better understand the content of the paper and reduce the need to frequently consult the reference section. Thank you again for your valuable feedback, which has greatly contributed to improving the readability and clarity of the manuscript.

---

2 Lines 69-74: I don't think this should be listed as contribution. On one hand, the first paragraph describes the experimental results, and on the other hand the use of accuracy and loss curves, and heatmaps are all pre-existing techniques for analyzing model's performance and understanding how the model works (e.g. heatmaps are done using the Grad-CAM method described in reference [46]).

Author response:

Thank you for your thorough review of our paper and valuable suggestions. Regarding the issue you raised in lines 69-74 about the contributions, we completely agree with your point. The comparison of the experimental results is a key evaluation metric for knowledge distillation methods, and therefore, we have revised the contributions section to include a direct comparison with SOTA methods. We agree with your suggestion that the accuracy curve, loss curve, and heatmap should not be listed as contributions. These are part of model performance analysis and should not be considered innovations in this paper. We have made the following revisions to the revised manuscript:

Extensive comparative experiments demonstrate the superior performance of the proposed THFKD across various datasets and network architectures. For instance, on Tiny-ImageNet, THFKD achieves a 0.71% improvement over state-of-the-art (SOTA) methods.

---

3 Lines 105-109: The authors indicate that "... the student model is susceptible to noise in the early stages of training...". However, they do not provide an alternative to tackle this problem, or at least it is not clear to me. If they provide an alternative, please better clarify it in the manuscript. Note that the authors do propose alternatives to SOTA methods for the other two subsections presented in "Related work", namely: "Knowledge distillation" and "Multi-branch

architectures and feature fusion".

Author response:

Thank you for your thorough review of our paper and valuable suggestions. Regarding the issue you raised in lines 105-109 about the 'student model being easily influenced by noise during the early stages of training,' we have provided a solution in the paper and further clarified the relevant content in the revised manuscript.

In the 'Two-Stage Optimization and Training' section of the Methods part, we explain in detail that during the early stages of training, the parameters of the student model are not yet stabilized and are prone to being affected by noise. Therefore, the initial value of the weight is set to a relatively small magnitude, and the weight is gradually increased over a predetermined period to smoothly guide the student model's optimization. In Equation 12, the loss term dominates, enabling the pre-trained teacher model to provide more comprehensive guidance in the initial phases.

We validated the effectiveness of the two-stage optimization strategy in the Ablation Experiment section under 'Effect of loss in different stages.' The experimental results show that by adjusting the loss weight appropriately, the stability of the student model in the early training stages improves, and the final classification accuracy is also enhanced.

---

4 Lines 145-150: I suggest improving the namings because:

a) the loss in (5) is referred to as "knowledge distillation";

b) the first paragraph in line 148 refers names "distillation loss" the K-L divergence loss L_{kl}. However, my first thought was that it is referring to L_{kd} because its name is "knowledge distillation" and L_{kl} is simply referred to as "loss" on line 146;

c) the loss in (2) is called "cross-entropy loss" therein, but on line 149 is called "label loss".

My suggested workaround is:

a) use "distillation loss" instead of just "loss" on line 146;

b) use "cross-entropy loss" instead of "label loss" on line 148.

Author response:

Thank you for your thorough review of our paper and valuable suggestions. We have made the revisions as per your suggestion: in line 146, 'loss' has been changed to 'distillation loss,' and in line 148, 'label loss' has been replaced with 'cross-entropy loss.

---

5 Fig. 2: It is not clear what the "high-level layers" are. Perhaps the position of the label "high-level layers" should be improved to clarify this.

Author response:

Thank you for your thorough review of our paper and valuable suggestions. Based on your feedback, we have revised Figure 2 to address the issue you raised about the unclear placement of the 'High-level Layers' label.

---

6 In expression (12) we read L_{cl} in the second term but it should be L_{el}.

Author response:

Thank you for your thorough review of our paper and valuable suggestions. Regarding the issue you raised about the second term in expression (12), we confirm that the formatting of the equation is correct. Equation (11) is $L_{cl} = L_{el} + L_{fl}$, and thus the total loss in Equation (14) is

$$L_{total} = \sum_{b=1}^{B} L_{ce}^{b} + w(i)(L_{cl} + L_{tg})$$

---

7 Following expression (12) we read "Eq. 14", but I it seems it should be "Eq. 12".

Author response:

Thank you for your thorough review of our paper and valuable suggestions. Regarding the issue you raised about the incorrect equation numbering, we confirm that after expression (12), the original text incorrectly refers to 'Equation (14),' but it should actually refer to 'Equation (12).' We have corrected the equation numbering in the corresponding sections of the text.

---

8 Following expression (12) we read "When E = i, ...". Since "E" is a constant and "i" is the variable (the epoch), it is more natural and intuitive to write "When i = E, ...".

Author response:

Thank you for your thorough review of our paper and valuable suggestions. Regarding the issue you raised about the expression 'when $E = i$' after expression (12), we completely agree with your point. E is a constant, and i is a variable (epoch), so a more natural and intuitive expression should be 'when $i = E$.' We have made this correction in the revised manuscript based on your suggestion:

When $i = E$, the period ends, and the weight is set to 1.

---

9 Line 233: Why is the first dimension of the concatenated logits 128? Please justify.

Author response:

Thank you for your thorough review of our paper and valuable suggestions. Regarding the issue you raised in line 233 about the '128' dimension, we confirm that '128' refers to the batch-size. To avoid causing unnecessary confusion for the readers, we have removed the description of this dimension in the revised manuscript:

Taking ResNet32 on CIFAR-100 as an example, the shape of the concatenated logits of all branches is 100×4 (where 4 is the number of branches B), and the loss for each branch is computed along the branch dimension.

---

10 Lines 258: It seems the weight decay should be 5E-4, not "5x10 - 4" as is currently written.

Author response:

Thank you for your thorough review of our paper and valuable suggestions. Regarding the issue you raised in line 258 about the representation of weight decay, we have revised it from '5x10 - 4' to '5E-4' based on your suggestion in order to conform to the standard scientific notation.

---

11 Table 1: How are methods in the rows and architectures in the columns sorted? By performance? By invention year? I suggest using a sensible criterion for sorting both rows and columns and indicate that criterion in the caption.

Author response:

Thank you for your thorough review of our paper and valuable suggestions. Regarding the issue you raised about the row (method) and column (architecture) ordering in Table 1, the ordering of our methods and architectures follows the arrangement commonly used in mainstream knowledge

distillation methods, such as CRD and ReviewKD.

---

12 Lines 291-292: what does it mean "Eq. 11: cl=fl+el." in the sentence "Different combinations of loss significantly impact classification accuracy, as shown in Eq. 11: cl=fl+el."?

Author response:

Thank you for your thorough review of our paper and valuable suggestions. We confirm that this misunderstanding was caused by the formula not being italicized. We have corrected the formula as per your suggestion, i.e., 'Equation 11: $L_{cl} = L_{fl} + L_{el}$.

---

13 Line 347: A `%` sign is missing after 71.14.

Author response:

Thank you for your thorough review of our paper and valuable suggestions. We have made the revisions in the revised manuscript.

---

14 Lines 347-349: The statement seems to be partially incorrect as OKDDip's performance doesn't fall below the ResNet32 performance of 71.14%, according to Table 5.

Author response:

Thank you for your thorough review of our paper and valuable suggestions. The content in lines 347-349 is not expressed clearly enough. We have rewritten this section in the revised manuscript. The specific revision is as follows:

We compared the online knowledge distillation methods based on multi-branch models, including CL, ONE, and OKDDip(all configured with four branches during training), with AFID, which employs dual independent models. As shown in Table 5, the first row presents the baseline classification accuracies of the high-parameter models ResNet8x4 and WRN16-2, which are 72.50% and 73.26%, respectively, both higher than the 71.14% accuracy of ResNet32. However, when trained using CL, ONE, and OKDDip, the classification accuracies of these high-parameter models (ResNet8x4 and WRN16-2) decrease compared to ResNet32 (72.56% > 61.81% and 72.43%; 73.50% > 61.66% and 72.86%; 74.32% > 72.75% and 73.96%). Notably, in the AFID, the independent model training approach does not exhibit such accuracy degradation (74.05% < 74.56% and 74.94%). In contrast, THFKD shows a 1.09% improvement over OKDDip when using ResNet8x4 and a 1.37% improvement over AFID when using WRN16-2 while avoiding the phenomenon where high-parameter models (ResNet8x4 and WRN16-2) underperform ResNet32 in classification accuracy. This demonstrates that THFKD adapts to various model architectures and effectively mitigates the problem of homogenization.

Table4.Compare the results of the OKD method on CIFAR-100.

Student	ResNet32	ResNet8x4	WRN16-2
	71.14	72.50	73.26
CL	72.56	61.81	72.43
ONE	73.50	61.66	72.86
OKDDip	74.32	72.75	73.96

AFID	74.05	74.56	74.94
THFKD	**75.41**	**75.82**	**76.31**

15 Fig 5:

a) Please correct the x labels to read "Accuracy curve" and "Loss curve".

b) Why is the ResNet32-L_{tg} Loss curve not plotted as is the case for the "Accuracy curve"?

Author response:

Thank you for your thorough review of our paper and valuable suggestions. The x-axis label in Figure 5 should be 'epoch,' as the figure displays the accuracy and loss values for each epoch. We have clarified this in the subfigure captions as 'Accuracy curve' and 'Loss curve' to help readers understand the content of the curves. Additionally, we have added the loss curve for ResNet32- L_{tg} in Figure 5 of the revised manuscript.

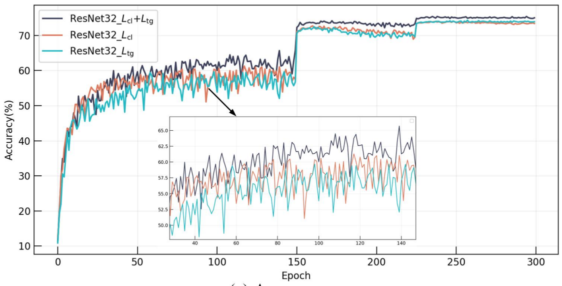

(a) Accuracy cure

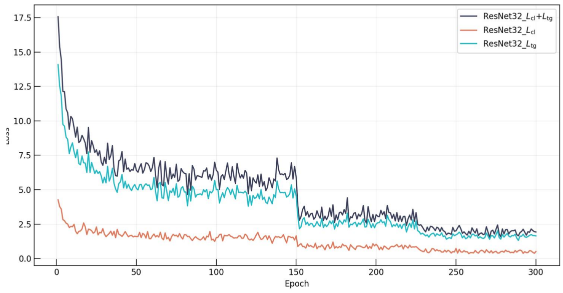

(b) Loss cure

16 Line 372: "When the parameter is less than or equivalent to the teacher model".. What does this mean? And what parameter are we talking about? Perhaps you meant "number of parameters" instead of "parameter"?

Author response:

Thank you for your thorough review of our paper and valuable suggestions. There is indeed a lack of clarity in this part. The revision is as follows:

When the parameter value of the ensemble model is less than or equal to the teacher model's parameter value, the ensemble's predictions achieve higher accuracy than those made by the teacher model.

17 Table 6: What does "MB" stand for in "parameters(MB)"?

Author response:

Thank you for your thorough review of our paper and valuable suggestions. Regarding the meaning of 'MB' in 'parameters(MB)' in Table 6, we confirm that 'MB' stands for 'Megabytes,'

which is used to measure the memory size occupied by the model parameters.

---

18 Lines 375-380:

a) The title says "different capacities" and the second sentence says "different capabilities". Is this correct or should "capabilities" be "capacities"?

b) What does it mean "the performance of the four branches gradually improves"? It improves with what? Perhaps you meant "Performance gradually improves as the number of branches improves"?

Author response:

Thank you for the reviewer's careful correction. The term 'abilities' should be used here to emphasize the differences in performance across the different branches. The revised content is as follows:

Branches with different abilities

THFKD's performance in the 300th epoch is shown under three model architectures. As shown in Table 7, the performance of the four branches gradually improves as the number of parameters increases (with the channel count in the auxiliary blocks growing multiplicatively), demonstrating differences in abilities. This provides a foundation for diversifying collaborative learning targets, allowing THFKD to meet varying precision requirements and enabling more flexible deployment.

---

19 Fig. 8: "Branch" is spelled incorrectly as "Branach".

Author response:

Thank you for your thorough review of our paper and valuable suggestions. Regarding the spelling error of 'Branch' as 'Branach' in Figure 8, we have corrected it to 'Branch' based on your suggestion.

Reviewer #6:

1.The cited research papers are mostly older than the year 2022. Hence, there is a space for comparison with the recent literature. There are some recent research articles related to the subject area covered in this research, like:

Gou, Jianping, Yu Chen, Baosheng Yu, Jinhua Liu, Lan Du, Shaohua Wan, and Zhang Yi. "Reciprocal teacher-student learning via forward and feedback knowledge distillation." IEEE transactions on multimedia (2024).

Guo, Wei, Xiang Li, and Ziqian Shen. "A lightweight residual network based on improved knowledge transfer and quantized distillation for cross-domain fault diagnosis of rolling bearings." Expert Systems with Applications 245 (2024): 123083.

Zhang, Sha, Jiajun Deng, Lei Bai, Houqiang Li, Wanli Ouyang, and Yanyong Zhang. "Hvdistill: Transferring knowledge from images to point clouds via unsupervised hybrid-view distillation." International Journal of Computer Vision 132, no. 7 (2024): 2585-2599.

Author response:

Thank you for your thorough review of our paper and valuable suggestions. Regarding the timeliness of the cited references and the comparison with the latest research, we have carefully analyzed the three papers you recommended and provide the following explanations:

Gou et al. (2024). This paper proposes a teacher-student mutual learning framework based on forward and feedback knowledge distillation. It does not compare with mainstream knowledge

distillation methods, and the network architecture used is different, so a fair comparison cannot be made. We have cited this paper in the relevant section.

Guo et al. (2024). This paper focuses on the application of lightweight residual networks in cross-domain fault diagnosis, which is a domain-specific study and is not directly related to the image classification task of this paper.

Zhang et al. (2024). This paper studies cross-modal knowledge distillation from images to point clouds, and its research area and methods differ significantly from the knowledge distillation framework in this paper.

In Table 2 of the revised manuscript, we have added a comparison with the latest 2024 paper, 'A new similarity-based relational knowledge distillation method.'

2.The reported accuracy of 69.64% should also be compared to the mentioned recent state-of-the-art (SOTA) systems.

Author response:

Thank you for your thorough review of our paper and valuable suggestions. Based on your feedback, we have added a comparison with the latest 2024 paper, 'A new similarity-based relational knowledge distillation method' (NRKD), in the revised manuscript.

Teacher	Student	KD[11]	AT[26]	CRD[23]	AFD[41]	NRKD[30]	THFKD
66.84	65.14	67.66	67.76	67.94	68.10	68.93	69.64

3.The accuracy is reported as the only performance metric in the manuscript. Other performance parameters like training/testing time may also be reported and compared with recent SOTA.

Author response:

Thank you for your thorough review of our paper and valuable suggestions. Regarding the issue you raised about reporting other performance metrics (such as training/testing time), we would like to explain as follows:

In knowledge distillation, mainstream research typically uses classification accuracy as the core performance metric, as it is the most direct and important standard for evaluating model performance. Additionally, the testing time is usually consistent when testing with the same network architecture because mainstream methods do not introduce additional computational costs during the inference stage. We reviewed relevant knowledge distillation papers and found that these studies do not report training/testing time. Therefore, we are unable to make an effective comparison of training/testing time with existing literature.

4.More graphs may be included in the manuscript explaining the behavior of Performance Metrics for the proposed system.

Author response:

Thank you for your thorough review of our paper and valuable suggestions. We have added ablation experiments on the depth and width of the heterogeneous branches further to analyze the impact of heterogeneous branches on model performance. The specifics are as follows:

(1)**Impact of temperature parameter** τ

The temperature parameter τ has a notable impact on the performance of the THFKD method. As shown in Table 5, the highest accuracy (75.41%) is achieved when $\tau = 3$, making it the optimal setting. A lower temperature ($\tau = 2$) may result in insufficient soft target information,

limiting the learning capability of the student model. On the other hand, as the temperature increases to $T=4$ and $T=5$, the performance gradually declines, indicating that excessively high temperatures may overly smooth the probability distribution, leading to the loss of critical information. Therefore, $T=3$ represents a balanced temperature that maintains sufficient information while avoiding excessive smoothing, providing effective guidance for the student model.

Table 5. Effect of temperature parameter T

T	2	**3**	4	5
THFKD	74.29	**75.41**	74.93	74.72

(2)To analyze the impact of heterogeneous branches (in terms of width and depth), we conducted experiments on the ResNet32 model by varying the number of auxiliary blocks and the channel size. Specifically, the number of auxiliary blocks was set to 0–2(NA = {0,1,2}), where NA = 0 indicates that each branch has the same structure without auxiliary blocks, and NA = 1 corresponds to the original experimental setting of THFKD. The channel size of the auxiliary blocks was set to either 1× or 2× the number of channels in the high-level layers (CA = { $C\times1$, $C\times2$ }), indicating that each branch has the same capacity. As shown in Table 8, the experimental results demonstrate that the number of auxiliary blocks (NA) and the channel size (CA) impact model performance differently.

First, regarding the number of auxiliary blocks (NA), the homogeneous branches without auxiliary blocks (NA = 0) exhibited the lowest accuracy (74.3%–74.6%), indicating that the model's feature representation and diversity were constrained. When NA =2, the accuracy of each branch improved (74.8%–75.0%); however, the performance of the more complex Branch-4 was inferior to that of Branch-3. This suggests that continuously increasing the number of auxiliary blocks does not necessarily enhance performance, and an overly complex structure may hinder model optimization. In contrast, the THFKD setting (NA = 1) achieved the best performance (75.0%–76.6%), with branch performance progressively improving, thereby validating the rationality of the heterogeneous branch design.

Regarding the impact of channel size (CA), when the channels were doubled (CA = $C\times2$), the accuracy of Branch-2 and Branch-3 increased to 75.77% and 75.90%, respectively. However, the accuracy of Branch-1 is similar under both CA = $C\times1$ and CA = $C\times2$, indicating that uniform channel expansion (i.e., identical auxiliary blocks) led to branches with similar abilities, failing to capture diverse feature representations. Consequently, the dynamic knowledge lacked sufficient diversity. The performance of THFKD (75.03%–76.61%) further confirms the effectiveness of the heterogeneous branch design.

Table.8 Accuracy(\%) of branches with different abilities on CIFAR-100.

B	Branch-1	Branch-2	Branch-3	Branch-4
NA=0	74.36	74.49	74.33	74.65
NA=2	74.83	75.02	75.64	75.06
CA=$C\times1$	74.51	74.71	75.16	75.15
CA=$C\times2$	74.44	75.77	75.90	75.74
THFKD(NA=1)	75.03	75.36	75.20	76.61

5.Conclusions should precisely address the claimed contributions made at the end of the Introduction section.

Author response:

Thank you for your thorough review of our paper and valuable suggestions. We have revised the conclusion and contributions sections based on your feedback:

contributions:

1. We propose a novel knowledge distillation training framework that divides the training process into two stages, enabling the student model to effectively learn comprehensive static knowledge and diverse dynamic knowledge, rather than relying exclusively on the teacher model.
2. We introduce heterogeneous branches and a Progressive Feature Fusion (PFF) module, which endows the branches with distinct abilities while efficiently fusion differentiated feature representations, thereby enhancing the diversity of dynamic knowledge.
3. Extensive comparative experiments demonstrate the superior performance of the proposed THFKD across various datasets and network architectures. For instance, on Tiny-ImageNet, THFKD achieves a 0.71\% improvement over state-of-the-art (SOTA) methods.

Conclusion:

We propose a novel Two-stage optimization based on heterogeneous branch fusion for knowledge distillation (THFKD). In traditional knowledge distillation, the student model is solely guided by the fixed knowledge of the teacher model, which lacks the ability to supplement or expand upon this knowledge, thereby limiting the student's generalization capability. In contrast, our student model leverages heterogeneous branches and a Progressive Feature Fusion (PFF) module to generate collaborative learning objectives, enabling the transfer of diversified dynamic knowledge, rather than relying exclusively on the teacher model. During training, the dynamic knowledge is combined with the static knowledge from the pre-trained teacher, while the two-stage optimization strategy provides appropriate knowledge at different training stages, enhancing the student model's learning efficiency and generalization ability. Extensive experiments were conducted on both standard datasets and long-tailed datasets, demonstrating that THFKD achieves superior performance compared to state-of-the-art methods. This demonstrates its broad application potential in image classification tasks. Additionally, we provide an analysis and visualization to further understand the underlying mechanisms of the proposed method. However, our method also exhibits certain limitations. THFKD performs slightly below existing approaches on lightweight models such as ShuffleNet, and the range of model combinations utilized is not yet comprehensive. In future work, we aim to further investigate these limitations and extend THFKD to other domains, such as object detection and image segmentation, to explore its broader applicability.

6.English language mistakes may be checked using human experts or specialized language software.

Author response:

Thank you for your thorough review of our paper and valuable suggestions. We have followed

your advice and reviewed and improved the grammar, spelling, and sentence structure throughout the manuscript.
