# Supplementary material for: Two-stage optimization based on heterogeneous branch fusion for knowledge distillation
Source: PLoS One. 2025 Jul 2;20(7):e0326711. doi: 10.1371/journal.pone.0326711 (PMC12221028; doi:10.1371/journal.pone.0326711)
Supplement: Attachment — Submitted filename: Review comments R1.pdf [file pone.0326711.s004.pdf]

Thanks to the authors for their careful revision, some issues need to be corrected further to improve the quality and readability of this article.

Innovativeness and Contributions

1. Elaborate on the Unique Advantages of the Innovations (Paragraph: Lines 66-69)

The authors propose heterogeneous branch structures and a progressive feature fusion module but need to provide a more detailed comparison with existing technologies. For instance, in lines 66-69, the authors mention, "We propose a novel knowledge distillation training framework," yet the specific differences between this framework and existing two-stage training methods (such as conventional KD and OKD) are not adequately highlighted.

Experimental Design and Results Analysis

2. Supplement Comparative Experiments with the Latest Research (Section: Experimental Results)

In the experimental results section (e.g., Tables 1 and 2), the authors primarily compare their method with classical approaches such as KD, FitNet, and CRD. It is suggested to supplement the experiments with comparisons to the latest knowledge distillation methods from 2023 to 2025, particularly on large-scale datasets like Tiny-ImageNet. For example, the authors could reference the following high-quality literature published in IEEE Transactions:

- "A Lightweight Residual Network Based on Improved Knowledge Transfer and Quantized Distillation for Cross-Domain Fault Diagnosis of Rolling Bearings" (Expert Systems with Applications, 2024)
- "Reciprocal Teacher-Student Learning via Forward and Feedback Knowledge Distillation" (IEEE Transactions on Multimedia, 2024)
- "Hvdistill: Transferring Knowledge from Images to Point Clouds via Unsupervised Hybrid-View Distillation" (International Journal of Computer Vision, 2024)

3. In-Depth Analysis of the Intrinsic Reasons for Experimental Results (Section: Table 3 and Related Analysis)

In Table 3, THFKD achieves an accuracy of 58.1% on the CIFAR100-LT dataset, but the reasons for this superior performance on long-tailed datasets are not thoroughly explained. It is recommended to add an analysis discussing how heterogeneous branches and progressive feature fusion assist the model in better learning the features of tail classes under long-tailed distributions. For instance, the authors could combine Grad-CAM visualization results to demonstrate how the model focuses on the key feature regions of tail classes.

4. Add an Analysis of the Trade-off Between **Model Complexity** and Performance (Section: Experimental Settings)

In the experimental settings section (lines 313-324), the authors mention the use of different model architectures but do not delve into the trade-off between model complexity and performance. It is suggested to add experiments showcasing the performance comparison between THFKD and other methods under different computational resource constraints (such as varying GPU memory usages), as well as how to select the appropriate number of branches and auxiliary block configurations. For example, a table could be added to display the accuracy and

memory usage of different branch numbers (2, 3, 4, 5) on the CIFAR-100 and Tiny-ImageNet datasets.

Writing Quality and Expression

5. Optimize the Article Structure and Logical Flow (Section: Related Work)

In the related work section (lines 73-140), the authors list various knowledge distillation methods but lack a clear classification and organization. It is suggested to categorize the introduction according to “methods based on static knowledge,” “methods based on dynamic knowledge,” and “hybrid methods” to facilitate readers’ understanding of the characteristics of different methods and THFKD’s positioning. For example:

- Methods based on static knowledge: including conventional KD, FitNet, etc.
- Methods based on dynamic knowledge: including DML, ONE, OKDDip, etc.
- Hybrid methods: methods that combine static and dynamic knowledge, such as THFKD.

6. Refine Language Expression (Throughout the Article)

It is recommended that the authors refine the language throughout the article to ensure concise and clear expression. Additionally, it is suggested that the authors engage **English-native experts** or professional language editing services to further enhance the quality of the language.

7. **Code and Documentation Optimization**

Enrich and Optimize Code and Documentation (Section: GitHub Link)

The GitHub link has been adequately supplemented, but it is recommended to further enrich and optimize the code and documentation to assist readers in better and faster reproduction and citation of the work. For example:

- Provide detailed instructions in the README document on how to install dependencies, run the code, and reproduce experimental results.
- Offer example code and configuration files demonstrating how to use the THFKD method for training and testing.
- Add code comments explaining the functions of key modules and functions to help readers better understand the code implementation.
