# Supplementary material for: Two-stage optimization based on heterogeneous branch fusion for knowledge distillation
Source: PLoS One. 2025 Jul 2;20(7):e0326711. doi: 10.1371/journal.pone.0326711 (PMC12221028; doi:10.1371/journal.pone.0326711)
Supplement: Attachment — Submitted filename: Response to Reviewers.docx [file pone.0326711.s005.docx]

Dear Editors and Reviewers:

Thank you for your letter and for the reviewers’ comments concerning our manuscript. Those comments are valuable and very helpful for revising and improving our paper, as well as the guiding significance to our studies. We have carefully considered the comments and have made revision which is hoped to meet with approval. In order to highlight the changes that we have made, we additionally submit a marked version of the paper and revised portion which is marked in blue.

*Reviewer #1:*

*1.Elaborate on the Unique Advantages of the Innovations (Paragraph: Lines 66-69)*

*The authors propose heterogeneous branch structures and a progressive feature fusion module but need to provide a more detailed comparison with existing technologies. For instance, in lines 66-69, the authors mention, “We propose a novel knowledge distillation training framework,” yet the specific differences between this framework and existing two-stage training methods (such as conventional KD and OKD) are not adequately highlighted.*

**Author response:**

Thank you for your review and valuable suggestions. we have refined the contribution section at the end of the introduction. The specific modifications are as follows:

1. We propose a novel knowledge distillation training framework. Unlike conventional approaches, our framework enables the student model to learn not only from a pre-trained teacher model but also to acquire dynamic knowledge from heterogeneous branches. The training process consists of two stages: first establishing foundational cognition through comprehensive static knowledge, then introducing diversified dynamic knowledge to achieve synergistic optimization between static and dynamic knowledge.

*2.Supplement Comparative Experiments with the Latest Research (Section: Experimental Results)*

*In the experimental results section (e.g., Tables 1 and 2), the authors primarily compare their method with classical approaches such as KD, FitNet, and CRD. It is suggested to supplement the experiments with comparisons to the latest knowledge distillation methods from 2023 to 2025, particularly on large-scale datasets like Tiny-ImageNet. For example, the authors could reference the following high-quality literature published in IEEE Transactions:*

*"A Lightweight Residual Network Based on Improved Knowledge Transfer and Quantized Distillation for Cross-Domain Fault Diagnosis of Rolling Bearings" (Expert Systems with Applications, 2024)*

*"Reciprocal Teacher-Student Learning via Forward and Feedback Knowledge Distillation" (IEEE Transactions on Multimedia, 2024)*

*"Hvdistill: Transferring Knowledge from Images to Point Clouds via Unsupervised Hybrid-View Distillation" (International Journal of Computer Vision, 2024)*

**Author response:**

Thank you for this valuable suggestion. We agree that incorporating comparisons with more recent knowledge distillation methods is important to demonstrate the competitiveness of our approach. In the revised manuscript, we have updated Tables 2 to include results from state-of-the-art distillation methods FFKD published 2024, on the Tiny-ImageNet dataset：

Table2. Accuracy(%) of KD Methods on Tiny-ImageNet. ResNet34-ResNet18.

| Teacher | Student | KD[11] | AT[26] | CRD[23] | AFD[41] | FFKD[42] | NRKD[30] | THFKD |
| --- | --- | --- | --- | --- | --- | --- | --- | --- |
| 66.84 | 65.14 | 67.66 | 67.76 | 67.94 | 68.10 | 68.51 | 68.93 | 69.64 |

[42] *Reciprocal Teacher-Student Learning via Forward and Feedback Knowledge Distillation" (IEEE Transactions on Multimedia, 2024)*

*3.In-Depth Analysis of the Intrinsic Reasons for Experimental Results (Section: Table 3 and Related Analysis)*

*In Table 3, THFKD achieves an accuracy of 58.1% on the CIFAR100-LT dataset, but the reasons for this superior performance on long-tailed datasets are not thoroughly explained. It is recommended to add an analysis discussing how heterogeneous branches and progressive feature fusion assist the model in better learning the features of tail classes under long-tailed distributions. For instance, the authors could combine Grad-CAM visualization results to demonstrate how the model focuses on the key feature regions of tail classes.*

**Author response:**

Thank you for your review and valuable suggestions. We have supplemented the explanation for the superior performance of THFKD on long-tailed datasets in Table 3. The specific modifications are as follows:

A long-tailed dataset has a highly imbalanced sample distribution, where the number of samples in a few categories is significantly larger than that in most categories. As shown in Table3, although THFKD is not explicitly designed for long-tail classification, it has demonstrated strong classification performance on the long-tailed dataset CIFAR100-LT. The heterogeneous branches designed in this paper allow each branch to specialize in different categories and data characteristics, thereby enhancing the model’s ability to capture information from tail classes. Building on this architecture, our progressive feature fusion module enables effective integration of differentiated features, while the knowledge distillation mechanism facilitates knowledge sharing and complementarity specifically for tail categories. This indicates that the student model is capable of learning comprehensively across all categories, avoiding bias toward the more frequent classes in the early stages of training, and further optimizing the model with diversified knowledge to enhance its generalization ability, thereby improving the classification performance of the model on the tail categories.

*4.Add an Analysis of the Trade-off Between Model Complexity and Performance (Section: Experimental Settings)*

*In the experimental settings section (lines 313-324), the authors mention the use of different model architectures but do not delve into the trade-off between model complexity and performance. It is suggested to add experiments showcasing the performance comparison between THFKD and other methods under different computational resource constraints (such as varying GPU memory usages), as well as how to select the appropriate number of branches and auxiliary block configurations. For example, a table could be added to display the accuracy and memory usage of different branch numbers (2, 3, 4, 5) on the CIFAR-100 and Tiny-ImageNet datasets.*

**Author response:**

We sincerely appreciate the reviewers' valuable suggestions regarding the model complexity analysis. Due to limited available equipment and GPU resources, we are currently unable to conduct experiments under different computational constraints. We fully agree that performance comparisons under varying resource limitations would be valuable, and we will make every effort to acquire additional hardware in the future to conduct these experiments.We have provided ablation studies on both the number of branches and auxiliary blocks in Figure 7 and Table 9, respectively.

*5.Optimize the Article Structure and Logical Flow (Section: Related Work)*

*In the related work section (lines 73-140), the authors list various knowledge distillation methods but lack a clear classification and organization. It is suggested to categorize the introduction according to “methods based on static knowledge,” “methods based on dynamic knowledge,” and “hybrid methods” to facilitate readers’ understanding of the characteristics of different methods and THFKD’s positioning. For example:*

*Methods based on static knowledge: including conventional KD, FitNet, etc.*

*Methods based on dynamic knowledge: including DML, ONE, OKDDip, etc.*

*Hybrid methods: methods that combine static and dynamic knowledge, such as THFKD.*

**Author response:**

Thank you for your valuable suggestions regarding the methodology section. As highlighted by the reviewer, a well-structured taxonomy can better elucidate the methodological evolution and our contribution.

Adopting the three-level classification system as suggested, existing methods have been reorganized into:

Methods based on static knowledge (Traditional KD, FitNet, etc.)

Methods based on dynamic knowledge (DML, ONE, OKDDip, etc.)

Multi-branch architectures and feature fusion

Terminology Retention Statement:

We retain "Multi-branch architectures and feature fusion" as the third category title because this terminology more precisely reflects the core innovation of our method.

*6.Refine Language Expression (Throughout the Article)*

*It is recommended that the authors refine the language throughout the article to ensure concise and clear expression. Additionally, it is suggested that the authors engage English-native experts or professional language editing services to further enhance the quality of the language.*

**Author response:**

Thank you for your thorough review of our paper and valuable suggestions. We have followed your advice and reviewed and improved the grammar, spelling, and sentence structure throughout the manuscript.

*7.Code and Documentation Optimization*

*Enrich and Optimize Code and Documentation (Section: GitHub Link)*

*The GitHub link has been adequately supplemented, but it is recommended to further enrich and optimize the code and documentation to assist readers in better and faster reproduction and citation of the work. For example:*

*Provide detailed instructions in the README document on how to install dependencies, run the code, and reproduce experimental results.*

*Offer example code and configuration files demonstrating how to use the THFKD method for training and testing.*

*Add code comments explaining the functions of key modules and functions to help readers better understand the code implementation.*

**Author response:**

Thank you for your thorough review of our paper and valuable suggestions. We have provided detailed code execution and experimental result reproduction instructions in the README documentation, along with code comments explaining key modules and loss functions, to facilitate readers' understanding of the implementation.

Reviewer #3:

*1.Clarity of Progressive Feature Fusion (PFF) Module: In the description of the PFF module, please elaborate on the exact role/calculation of the scalar parameter 'g' mentioned in Eq. 8. How is 'g' determined or learned?*
**Author response:**

Thank you for your review and valuable suggestions. A detailed explanation of the scalar parameter g is as follows:

In the fusion process described in Equation (8), $g$ as a balancing factor, dynamically weighting the contributions of the current branch feature $F_{b}$ and the fused features $F_{b}^{m-1}$ from the previous layer, with parameter $g$ is a scalar parameter with a value range of 0 to 1, when $g$ approaches 1, the current branch features dominates the fusion output, when $g$ approaches 0, the fused features from the previous level receive higher weight. In our method, $g$ is set to 0.5, ensuring equal importance of both current branch features and prior fused features. We have provided a more comprehensive explanation of this mechanism in the revised manuscript.

Reviewer #5:

*1.In the abstract we read "THFKD effectively improves...". I recommend changing the whole sentence starting at "Experiments results ... generalization ability" to "Although no tests of statistical significance were carried out, our experimental results on standard datasets (CIFAR-100, Tiny-ImageNet) and long-tail datasets (CIFAR100-LT) suggest that THFKD may slightly improve the student model’s classification accuracy and generalization ability."*

*The reason for this change is that the increase in accuracy is really marginal, as e.g. stated in the phrase following the above sentence, where the improvement in accuracy is quantified as just 0.71%. The improvement is so small that we cannot be certain that it is an actual signal, or it is something obtained just by chance, as no statistical analysis was carried out to confirm it.*

**Author response:**

Thank you for your important reminder regarding the rigor of experimental results presentation. We have fully adopted your suggestions and revised the abstract as follows:

Although no tests of statistical significance were carried out, our experimental results on standard datasets (CIFAR-100, Tiny-ImageNet) and long-tail datasets (CIFAR100-LT) suggest that THFKD may slightly improve the student model’s classification accuracy and generalization ability. For instance, using ResNet18 on the Tiny-ImageNet dataset, the accuracy reaches 69.64\%, a 0.71\% improvement over the state-of-the-art (SOTA).

*2.Line 176: The description of the "y" labels was improved to indicate they correspond to one-hot encoding of the actual label used to classify the images. However, I still suggest adding a short phrase to the sentence to make the definition of one-hot encoding complete, namely by adding the phrase after the comma in the following sentence: "with its corresponding one-hot label y = [y1, ..., yK], where y1, ..., yK \in {0, 1} and \sum_k {yk} = 1".*
**Author response:**

Thank you for your rigorous requirements regarding the mathematical notation definitions. We have made the following improvements to Line 176:

Given a dataset with $K$ classes, where $K\in(1,...,K)$, and a training sample $x$ with its corresponding one-hot label $y=[y_{1},...,y_{K}]$, where $y_{1},...,y_{K}\in\text{（}0,1\text{）}$ and $\sum_{k=1}^{K} y_{k}=1$, when $x$ is input into the student model, the output probability of the $b$-th branch for class $k$ is calculated as:

*3.Although no statistical analysis has been performed to quantify the significance of the differences in accuracy observed across methods, almost all mentioning to "a significant difference" were correctly removed from the text.*

*However, there is still one use of "significant" which I suggest replacing with a quantitative evaluation: in line 432 we read "THFKD exhibits significant performance improvement when the number of branches is set to 4." I suggest replacing this sentence with "THFKD exhibits a performance improvement of about 2% (from ~ 73% to 75%) when the number of branches is increased from 1 to 4.". Again, no statistical test was carried out to evaluate whether this increase in performance is statistically significant, this is the reason for removing the word "significant" and providing instead the actual quantitative change observed in the specific experiment carried out by the authors.*

**Author response:**

Thank you for your persistent suggestions regarding terminology refinement. We have implemented the following key improvements to Line 432:

We set the total number of branches $B$ to range from 2 to 6 to analyze the impact of branch quantity on performance. As shown in Fig.7, THFKD exhibits a performance improvement of about 2% (from 73% to 75%) when the number of branches is increased from 1 to 4.
*4.(1)Lines 448-475: I find the new quantitative analysis on the impact of heterogeneous branches a little "over-conclusive". As seen in Table 9, the differences in accuracy when varying parameters NA and CA is always VERY small: except for 5 values of the 20 values reported (namely 75.64, 75.77, 75.90, 75.74, 76.61), the difference between any of the accuracy values and the smallest accuracy for the "NA=0, Branch-1" case (= 74.36) is at most 1%. That is, the effect of branch heterogeneity doesn't seem to be very strong... The observed differences could just be noise. I don't find the conclusion in the last line ("The performance of THFKD (75.03%–76.61%) further confirms the effectiveness of the heterogeneous branch design.") really correct. The accuracy even decreases when going from branch-2 to branch-3 from 75.36 to 75.20, suggesting that no strong signal about accuracy improvement is present in the reported values...*

*I suggest thoroughly re-writing all three paragraphs in view of the above, and avoid conveying that there is a strong effect on accuracy by branch heterogeneity.*

*(2) Lines 450-451: The acronym "NA" is mentioned but not defined. The sentence should read: "Specifically, the number of auxiliary blocks (NA) was set to ...". The other two repetitions of the definition of NA (in lines 456 and 458) should be removed because NA is now defined on line 450.*

*(3) Same comment for the "CA" acronym applies as for the "NA" acronym: CA should be defined the first time "channel size" is mentioned, namely on line 453, and then be removed from their redefinition below (lines 456 and 468).*

**Author response:**

We sincerely appreciate your meticulous review of our experimental analysis and terminology standardization. We have rigorously implemented your suggestions through the following improvements:

To analyze the impact of heterogeneous branches in terms of width and depth, we conducted experiments on the ResNet32 model by varying the number of auxiliary blocks (NA) and their channel size (CA). Specifically, the number of auxiliary blocks (NA) was set to {0, 1, 2}, where NA = 0 indicates that each branch has the same structure without auxiliary blocks, and NA = 1 corresponds to the original experimental setting of THFKD. The channel size (CA) was set to either 1× or 2× the number of channels in the high-level layers (CA = $C\times1,C\times2$).

As shown in Table 8, although the absolute differences in accuracy are relatively small (mostly within 1%), some consistent trends can still be observed. Firstly, when NA=0, all branches perform poorly (ranging from 74.3% to 74.6%), which may be due to the lack of auxiliary structures limiting feature diversity. Introducing auxiliary blocks (NA=2) generally improves performance (e.g., Branch-3 reaches up to 75.64%), but the trend is not strictly monotonic—overly complex branches such as Branch-4 show a slight performance drop, possibly due to increased optimization difficulty. This suggests that simply stacking more modules does not guarantee better results, and moderate structural diversity may be more beneficial.

Impact of the number of channels (CA): Doubling the number of channels improves performance in some branches (e.g., Branch-2 and Branch-3 reach 75.77% and 75.90%, respectively), indicating that selective capacity expansion can enhance feature learning ability. However, for CA = $C\times1,C\times2$, the accuracy fluctuations of Branches 2–4 are relatively small (with the maximum and minimum accuracy differences being 0.45 and 0.16, respectively), which suggests that uniform channel expansion (i.e., using identical auxiliary blocks) leads to similar capabilities among branches, thereby reducing the diversity of dynamic knowledge. The best performance is observed under the THFKD configuration (NA=1, CA=$C\times1$), with Branch-1 achieving an accuracy of 75.03%. Although the performance improvement is modest, the accuracy variation among Branches 2–4 is relatively large (a maximum difference of 1.41), indicating that well-designed heterogeneous branches in both depth and width help promote diversity in feature representation.

*5.Conclusion: Although the authors removed their initially (apparently arbitrary) selection of the quantitative results to report from the Conclusion section, the new sentence is too vague, namely: "THFKD achieves superior performance compared to state-of-the-art methods". How much superior? I think the superiority is not strong enough and the level of noise in the accuracy results has not been evaluated. This lack of statistical analysis does not allow to write a conclusion that states that THFKD is superior to the SOTA methods. Please see my suggestions for modification of the abstract in item (1) above for a guideline on how to change this conclusion.*

**Author response:**

We sincerely appreciate your valuable suggestions for improving the conclusiveness of our findings. We have implemented the following enhancements to strengthen our conclusions:

We propose a novel Two-stage optimization based on heterogeneous branch fusion for knowledge distillation (THFKD). In traditional knowledge distillation, the student model is solely guided by the fixed knowledge of the teacher model, which cannot supplement or expand upon this knowledge, thereby limiting the student's generalization capability. In contrast, our student model leverages heterogeneous branches and a Progressive Feature Fusion (PFF) module to generate collaborative learning objectives, enabling the transfer of diversified, dynamic knowledge rather than relying exclusively on the teacher model. During training, the dynamic knowledge is combined with the static knowledge from the pre-trained teacher, while the two-stage optimization strategy provides appropriate knowledge at different training stages, enhancing the student model's learning efficiency and generalization ability. Although no tests of statistical significance were carried out, our experimental results on standard datasets (CIFAR-100, Tiny-ImageNet) and long-tail datasets (CIFAR100-LT) suggest that THFKD may slightly improve the student model’s classification accuracy and generalization ability. This demonstrates its broad application potential in image classification tasks. For instance, using ResNet110-ResNet32 on the CIFAR-100 dataset, the accuracy reaches 75.41%, a 1.52% improvement over the state-of-the-art (SOTA). Additionally, we provide an analysis and visualization to further understand the underlying mechanisms of the proposed method. However, our method also exhibits certain limitations. THFKD performs slightly below existing approaches on lightweight models such as ShuffleNet, and the range of model combinations utilized is not yet comprehensive. In future work, we aim to investigate these limitations further and extend THFKD to other domains, such as object detection and image segmentation, to explore its broader applicability.
