# Supplementary material for: Two-stage optimization based on heterogeneous branch fusion for knowledge distillation
Source: PLoS One. 2025 Jul 2;20(7):e0326711. doi: 10.1371/journal.pone.0326711 (PMC12221028; doi:10.1371/journal.pone.0326711)
Supplement: Attachment — Submitted filename: Reviewer comment.pdf [file pone.0326711.s001.pdf]

The main content of this paper is focused on a two-stage optimization approach based on heterogeneous branch fusion for knowledge distillation. The authors propose a collaborative learning target that consists of Fusion Logits obtained from fused features and Ensemble Logits derived from the integration of branch logits. They introduce a multi-branch architecture where each branch shares parameters in the low-level layers but has independent parameters in the high-level layers. The authors also design a progressive feature fusion module to enhance the utilization of features and improve the representational capability of the model. Experimental results on standard datasets such as CIFAR-100, Tiny-ImageNet, and CIFAR100-LT demonstrate that the proposed method significantly improves the classification accuracy and generalization ability of the student model.

However, there are some issues that need improvement:

1. The limitations of existing methods are suggested to be refined to highlight the value of the proposed method. Related work needs to be further organized rather than just listed.
2. The contributions of the paper should be stated more clearly and explicitly. The innovation points and contribution statements at the end of the introduction section are suggested to be further refined and condensed to highlight the core values of this paper.
3. More high-quality papers need to be added to further highlight the value of the proposed method, such as the introduction or related work chapters, including but not limited to the following papers:

   [1] Knowledge distillation: A survey[J]. *International Journal of Computer Vision*, 2021, 129(6): 1789-1819.

   [2] Learning from Human Educational Wisdom: A Student-Centered Knowledge Distillation Method[J]. *IEEE Transactions on Pattern Analysis and Machine Intelligence*, 2024, 46(6): 4188-4205.

   [3] Improving Knowledge Distillation via Head and Tail Categories[J]. *IEEE Transactions on Circuits and Systems for Video Technology*, 2024, 34(5): 3465-3480.

   and so on…
4. https://github.com/GitLpf/THFKD. The github URL provided by the author is missing the necessary code and other information. However, one of the most valuable contributions of this paper is the availability of their open-source code. Therefore, **it is strongly recommended that** the authors carefully organize the relevant de-sensitization data, code, and processing flow introduction into **github** or other **open-source** forms, and give them in the form of URL links in the **Abstract**, so as to enhance the influence of the text work and attract more interested readers to follow the work of this paper.
5. In the Section of Methodology, the two-stage optimization strategy and progressive feature fusion module should be described in more detail with mathematical formulations.
6. In the Section of Methodology, the loss functions and training procedure should be explained more thoroughly.
7. In the Section of Experiments, The experimental setup (datasets, model architectures, hyperparameters) should be described in more detail.
8. More ablation studies should be added to analyze the impact of different components (e.g.

fusion module, loss weights). Moreover, more tables are suggested to be added to visually present the comparison results of the performance before and after ablation.

9. The results should be analyzed in more depth, with insights into why the proposed method works well.
10. The results should be analyzed in more depth, with insights into why the proposed method works well.
11. In the Section of Conclusion, the conclusion should summarize the key findings and contributions. Limitations of the current work and potential future directions should be discussed.
12. The writing should be polished for clarity, grammar, and flow. Some sentences are overly long and could be broken up. Consistent terminology should be used throughout (e.g. "student model" vs "target model").
